# Whole-Genome Diversification Analysis of the Hornbeam Species Reveals Speciation and Adaptation Among Closely Related Species

**DOI:** 10.3389/fpls.2021.581704

**Published:** 2021-02-10

**Authors:** Zeyu Zheng, Ying Li, Minjie Li, Guiting Li, Xin Du, Hu Hongyin, Mou Yin, Zhiqiang Lu, Xu Zhang, Nawal Shrestha, Jianquan Liu, Yongzhi Yang

**Affiliations:** ^1^State Key Laboratory of Grassland Agro-Ecosystem, Institute of Innovation Ecology and School of Life Sciences, Lanzhou University, Lanzhou, China; ^2^CAS Key Laboratory of Tropical Forest Ecology, Xishuangbanna Tropical Botanical Garden, Chinese Academy of Sciences, Kunming, China

**Keywords:** speciation, adaptation, *Carpinus*, divergence, selection, CNV

## Abstract

Speciation is the key evolutionary process for generating biological diversity and has a central place in evolutionary and ecological research. How species diverge and adapt to different habitats is one of the most exciting areas in speciation studies. Here, we sequenced 55 individuals from three closely related species in the genus *Carpinus*: *Carpinus tibetana*, *Carpinus monbeigiana*, and *Carpinus mollicoma* to understand the strength and direction of gene flow and selection during the speciation process. We found low genetic diversity in *C. tibetana*, which reflects its extremely small effective population size. The speciation analysis between *C. monbeigiana* and *C. mollicoma* revealed that both species diverged ∼1.2 Mya with bidirectional gene flow. A total of 291 highly diverged genes, 223 copy number variants genes, and 269 positive selected genes were recovered from the two species. Genes associated with the diverged and positively selected regions were mainly involved in thermoregulation, plant development, and response to stress, which included adaptations to their habitats. We also found a great population decline and a low genetic divergence of *C. tibetana*, which suggests that this species is extremely vulnerable. We believe that the current diversification and adaption study and the important genomic resource sequenced herein will facilitate the speciation studies and serve as an important methodological reference for future research.

## Introduction

Speciation, the formation of new species, is a key evolutionary process that gives rise to biodiversity on Earth (Coyne and Orr, 2004; Bokma, 2010). Understanding how and why genomes diverge during speciation is fundamental to understanding how species evolve (Seehausen et al., 2014; Ma et al., 2017). The ideal speciation event should have complete genetic and geographic isolation (Bouman, 1995; Bokma, 2010), with no gene-flow between species, and is associated with natural selection and drift (Han et al., 2017). However, during the speciation process, we can always observe gene flow (Wang et al., 2019), which opposes the divergence between lineages (Hart, 2014). Hence, identifying and quantifying the gene flow between diverging lineages is critical for documenting the history of speciation (Faria et al., 2014; Seehausen et al., 2014). In recent years, the advances in high-throughput sequencing and computational biology (Nosil and Feder, 2012; Feder et al., 2013) have facilitated the study of population genomic divergence within and between closely related species adapted to different habitats. For example, the isolation-with-migration model, which is based on both likelihood and Bayesian frameworks, has been used to undertake joint estimation of multiple demographic parameters and to demonstrate speciation by analyzing gene-flow in sticklebacks (Nielsen and Wakeley, 2001; Carrara et al., 2007). The simulation-based framework has also been used to infer demographic parameters from site frequency spectrum (SFS) in African human populations (Excoffier et al., 2013). This is one of the most exciting areas in evolutionary biology, and it has important implications for enhancing our understanding of the origin of species (Bokma, 2010).

Hornbeam (*Carpinus* L., containing ∼50 species) is one of the most diverse genera within Betulaceae. The majority of closely related species in the genus diverged less than 5 Mya (Yang, 2018). Most hornbeams are medium-sized trees and distributed in the temperate regions of the northern hemisphere with wide adaptability to different environments. Hence, hornbeams may contribute substantially to global photosynthesis and carbon sequestration rates (Yang, 2018). Hornbeams also have important economic value. They are commonly used as urban street trees and building timber. *Carpinus* is an ideal system for studying recent speciation and diversification process (Li and Skvortsov, 1999; Lu et al., 2018; Yang, 2018). In the present study, we focused on the three species of the genus: *Carpinus monbeigiana* Hand.-Mazz, *Carpinus mollicoma* Hu, and *Carpinus tibetana* (Lu et al., 2018), because these three species show a very close resemblance in morphology and internal transcribed spacer (ITS) sequence (Lu et al., 2018). *C. monbeigiana* is distributed mainly in the central and northwestern forests of Yunnan at an altitude of approximately 1,700–2,800 m (Li and Skvortsov, 1999). *C. mollicoma* prefers stony hillsides and is mainly distributed in the southeastern part of Yunnan province and Mount Emei in Sichuan province, at an altitude of 1,000–1,700 m (Li and Skvortsov, 1999). *C. monbeigiana* usually grows up to 16 m, which is taller than *C. mollicoma*, which only grows up to 10 m (Li and Skvortsov, 1999). Both *C. monbeigiana* and *C. mollicoma* have oblong-lanceolate leaves, but *C. monbeigiana* has ovate and much larger leaves. *C. monbeigiana* also has shorter petiole and has comparatively less pubescent leaves. The infructescence and bract size of *C. monbeigiana* are also larger (Li and Skvortsov, 1999; Lu et al., 2018). *C. tibetana* is a recently discovered species distributed only in the Qinghai–Tibet Plateau (QTP) area. It exhibits a close relationship with *C. monbeigiana* (Lu et al., 2018) but has more lateral veins and nutlet ribs than *C. monbeigiana* and *C. mollicoma*. These three species can be clearly distinguished based on morphology (Lu et al., 2018).

Here, we sampled 13 *C. monbeigiana*, nine *C. mollicoma*, and 31 *C. tibetana* individuals (∼587 Gb raw data) for the population genomic analysis, which included investigations of population structure, demographic history, gene flow, selection, and divergence analysis at the whole-genome level. Based on these genomic data and analysis, we aimed to infer the relationship among these three species and estimate the strength and direction of gene flow and selection during the speciation process.

## Materials and Methods

### Sampling and Single Nucleotide Polymorphism Calling

**FIGURE 1:**
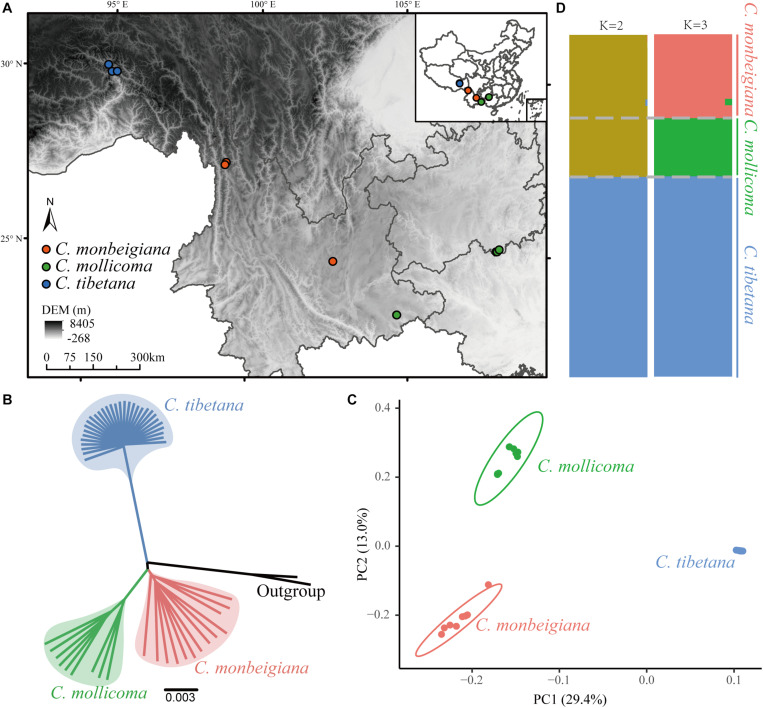
Phylogenetic and population genetic analyses. **(A)** Biogeographic locations of whole-genome-sequenced individuals, sampled for three species. **(B)** Phylogenetic tree constructed using the neighbor-joining method based on whole-genome consensus sequence. **(C)** PCA plots of SNP data for three species. **(D)** Population structure of tree species. Each vertical bar represents a single individual, and height of each color represents probability of assignment to that cluster.

Our dataset contained a total of 53 hornbeam samples, including 9 *C. mollicoma* from the southeastern Yunnan, 13 *C. monbeigiana* from the northwestern Yunnan, and 31 *C. tibetana* from the QTP region (Figure 1A). As *C. tibetana* has a very narrow habitat range in the QTP, those 31 samples were collected to represent this species. All the individuals were collected at least 5 km apart in the wild. We also sampled one *Carpinus cordata* Bl. and one *Ostrya japonica* Sarg. individuals as outgroup. A total of 55 samples were sequenced in this study (Supplementary Table 1). Total genomic DNA was isolated by the cetyl trimethylammonium bromide method (Liu et al., 2009) and then used to construct 250-bp insert pair-end libraries according to the Illumina manufacturer’s instructions using Ultra II DNA Library Kits for Illumina (NEB #7370L). Based on the Hiseq2500 platform, ∼8-Gb sequencing data were generated for each sample. Trimming adaptor of Raw data were through SCYTHE^1^ and then used SICKLE^2^ to remove low-quality (shorter than 50 bp or quality scores less than 20) reads. BWA-MEM (Li and Durbin, 2010) version 0.7.17 was used to align trimmed reads to the *Carpinus fangiana* reference genome (Yang et al., 2020) with default parameters. Finally, we used the MarkDuplicates, RealignerTargetCreator and IndelRealigner tools provided in Picard (McKenna et al., 2010), and GATK v3.8 (Van der Auwera et al., 2013) with default parameters to remove PCR duplications and rematch InDel regions.

For single nucleotide polymorphism (SNP) calling, we used GATK-HaplotypeCaller in GVCF mode to prevent biases in SNP calling accuracy between species for which there were different numbers of samples. After joint genotyping across samples (using GATK-GenotypeGVCFs), we performed strict filtering of each single-sample SNP and multi-sample SNPs to reduce false positives. The InDels obtained were filtered by GATK-VariantFiltration with the parameters “QD < 2.0 | | Read-PosRankSum < −20.0 | | FS > 200.0,” and for SNPs, the settings were “QD < 2.0 | | FS > 60.0 | | ReadPosRankSum < −8.0 | | MQ < 40.0 | | MQRankSum < −12.5 | | SOR > 3” (Ma et al., 2017). We also performed additional filtering steps to remove SNPs with: (1) quality score <50, (2) extremely high (> three-fold average depth), or extremely low (< one-third average depth) coverage, (3) SNPs at or within 5 bp of any indels, and (4) SNPs with more than two alleles in all samples. Samples with very close relationships might bias the accuracy of results. We, therefore, used identical by state distances estimated by PLINK (Chang et al., 2015) to identify their relationships. Samples with identical by state >0.9 were discarded. Thus, we removed one sample from within the *C. mollicoma* dataset.

### Phylogenetic Analysis and Population Structure Analyses

To analyze the phylogenetic relationship of the three species, we constructed a phylogenetic tree using the neighbor-joining (NJ) method with the whole-genome consensus sequence and SNP sites by ClustalW2 (Larkin et al., 2007). Among the phylogenetic tree building steps, *C. cordata* and *O. japonica* were considered as outgroups. Trees visualization was implemented in FigTree v1.4.4^3^. The branch support scores were calculated from 1,000 bootstraps of the NJ phylogenetic tree using Fastphylo (Khan et al., 2013) and then combined by PHYLIP (Felsenstein, 2005). Principal component analysis (PCA) of the three species was performed using the smartPCA program from the EIGENSOFT package v7.2.1 (Price et al., 2006). The population structure was inferred using ADMIXTURE v1.23 (Alexander et al., 2009), setting the putative number of populations (*K*) from 1 to 6. The optimum number of subgroups (*K*) was determined based on the minimum ADMIXTURE cross-validation error value (Alexander and Lange, 2011).

### Within-Population Diversity and Population Status

The genetic variation within the three species was estimated *via* theta pi (θ_π_) (Nei, 2019), which was divided into 50-Kb non-overlapping sliding windows and calculated by VCFtools (Danecek et al., 2011). Simultaneously, we computed the Tajima’s *D-*value (Tajima, 1989) as a test of neutrality to examine genomic evidence for population expansion or decline. Tajima’s *D* was also calculated by the 50-Kb non-overlapping window method. The estimation of heterozygous for each individual were computed using mlRho (Haubold et al., 2010) following default settings.

### Demographic History and Gene Flow Estimating

To determine whether *C. monbeigiana* or *C. mollicoma* has more gene-flow with *C. tibetana*, the ABBA–BABA test (also known as the *D* statistic) (Martin et al., 2015) was performed. We estimated the D and *f*_d_ statistic value using the whole population of *C. monbeigiana*, *C. mollicoma*, and *C. tibetana*, and using *C. cordata*, and *O. japonica* together as the outgroup (Supplementary Figure 1). We split the genome by 50 K window and calculated *D* and *f*_d_ statistics for each window. Finally, jackknife (Efron and Tibshirani, 1993) in R package (R Core Development Team, 2019), which is named “bootstrap,” was used with default parameters for inferring statistical difference. A positive *f*_d_ statistic value indicates the introgression from population *C. tibetana* to *C. monbeigiana*, whereas zero means no introgression.

To explore effective population size (*Ne*) history, we used the pairwise sequentially Markovian coalescent (PSMC) (Li and Durbin, 2011), which was a widely used method that showed high accuracy and widely used in plants and animals (Bai et al., 2018; Patton et al., 2019). During the PSMC analysis, *Ne* was inferred across 28 free atomic time intervals with the following parameters: −*N* 25 −*t* 15 −r 5 −*p* “4 + 25 × 2 + 4 + 6.” Consensus sequences were obtained using SAMtools and divided into non-overlapping 100-bp bins. Bases of low sequencing depth (less than a third of the average depth) or high depth (twice the average depth) were masked as recommended (Li and Durbin, 2011). Bootstrapping with 100 repetitions was done to estimate the variance of the simulated results. Approximately five individuals per species were selected to represent the whole population.

As the PSMC method cannot take into account the gene flow conditions, we further applied demographic inferences based on fitting models to folded SFS data. To obtain the SFS with more accuracy, we used Samtools to re-call all variant and non-variant sites after filtering out low-depth (<1/3 of mean depth) and low-quality (QD < 2) sites. The fastsimcoal package v2.6 (Excoffier et al., 2013) was used to infer the demographic history of *C. monbeigiana* and *C. mollicoma*. The generation time was set to 15 years, and mutations per site per year were set at 3.75 × 10^–8^ (Yang, 2018). We tested divergence with two-way, one-way, or without gene flow, and then, one population size changes per species were estimated (Supplementary Figure 2). All parameter estimates were obtained from 100 independent fastsimcoal runs, with 100,000 simulations per pseudolikelihood estimation and 40 cycles of the pseudolikelihood maximization algorithm. The model with optimal pseudolikelihood and Akaike’s information criterion was identified as the best model. Confidence intervals of parameter estimates were obtained by parametric bootstrapping with 100 bootstrap replicates per model. Residuals were calculated as the comparison of the observed site frequency spectra with the simulation based on the best demographic model.

### Genome-Wide Patterns of Differentiation and Selection

The allele-frequency-based approach *F*_ST_ was used to detect selective sweep region implemented in VCFtools with 50-Kb non-overlapping sliding windows. Only sliding windows with *z*-transformed *P*-values less than 0.05 were treated as significant. For selection analysis, we used an linkage disequilibrium-based approach cross-populations extended haplotype homozygosity (XP-EHH) (Sabeti et al., 2007) implemented in selscan (Szpiech and Hernandez, 2014). The XP-EHH analysis was based on phased haplotypes conducted using Beagle v4.1 (Browning and Browning, 2007). Because of the absence of an entirely constructed genetic map, genetic positions were assumed to be equivalent to physical positions (1 Mb = 1 cM), which have been used in soybean (Kim et al., 2019), dove-tree (Chen et al., 2020), pig (Ma et al., 2015; Diao et al., 2019), and sheep (Fariello et al., 2013). This transformation has been tested and found that it did not change the main linkage results (Ulgen and Li, 2005; Chen et al., 2020). We used a 50-Kb non-overlapping sliding window calculation. *C. monbeigiana* was used as the reference, and windows that did not own SNPs were filtered. The negative XP-EHH values suggest that selection occurred in the reference population (*C. monbeigiana*), whereas positive values indicated selection in the observed population (*C. mollicoma*). Only sliding windows with *z*-transformed *P*-values less than 0.05 were treated as significant.

Functional analysis of genes was carried out against the Gene Ontology (GO) database (Ashburner et al., 2000). Functional classification of GO categories for the entire gene set was performed using the InterProScan v5.36 (Jones et al., 2014) program with the PANTHER database (Mi et al., 2019). Enrichment analysis was performed with the R package TopGO (Alexa and Rahnenfuhrer, 2010; R Foundation, 2018) and visualized using the REVIGO (Supek et al., 2011) website.

### Identification of Copy Number Variants and Differentiated Genes

Nowadays, researchers focus not only on SNPs but also on another important source of genetic structure variation, i.e., copy number variants (CNVs), which are found to be an important genetic resource (Zhou et al., 2011; Iskow et al., 2012; Keel et al., 2016). We used the read depth-based approach implemented in Control-FREEC (Boeva et al., 2012) to estimate the integer copy numbers for each 10-kb window with a 2-kb step size across the entire genome. Control-FREEC has been widely used in copy number variant (CNV) annotation in rice (Ghouri et al., 2019), poplars (Lin et al., 2018), rape (Hua et al., 2018), and polar bears (Rinker et al., 2019). The parameters we used in Control-FREEC were: breakPointThreshold = 0.8, coefficientOfVariation = 0.062, degree = 3, telocentromeric = 0. Then, we calculated gene CNVs by using the 10-kb window CNVs. For genes contained by multiple windows, the average value was used.

To investigate copy number differentiation between *C. monbeigiana* and *C. mollicoma*, we used the *V*_ST_ (Redon et al., 2006; Rinker et al., 2019) measure, which is analogous to *F*_ST_ but is specific for multi-allelic genotype data such as CNVs. *V*_ST_ was calculated for 10-kb sliding windows and with a 2-kb step size across the whole genome. To minimize sampling bias within each population, we used a permutation test (Rinker et al., 2019) to compute the differentiation of each gene. For this, we randomly permuted all *C. monbeigiana* and *C. mollicoma* individuals and calculated a new *V*_ST_ for each gene, 1,000 times. Only genes with observed *V*_ST_ values above the maximum 99% confidence of 1,000 repeats were considered to be significant.

## Results

### Resequencing and Single Nucleotide Polymorphisms

A total of 587 Gb of clean reads were generated from whole-genome resequencing of 13 *C. monbeigiana*, 9 *C. mollicoma*, 31 *C. tibetana*, 1 *C. cordata*, and *Ostrya trichocarpa* samples from southwest China, with an average sequencing depth of 20.7X (Supplementary Table 1 and Figure 1A). Sequences were aligned to the *C. fangiana* reference genome. After SNP and InDel calling and filtering (see section “Material and Methods”), 13.8 million SNPs and 3.2 million InDels were detected. A total of 9.6 million SNPs were shared by all three species, and 4.8, 1.6, and 0.6 million SNPs were present only in *C. monbeigiana*, *C. mollicoma*, and *C. tibetana* populations, respectively (Supplementary Figure 3).

### Population Structure

To examine the genetic relationships among individuals, we constructed a phylogenetic tree using the NJ method with both the whole-genome SNP dataset (Supplementary Figure 4) and the whole-genome consensus sequences (Figure 1B). This phylogenetic analysis showed a clear divergent relationship among the three lineages: *C. tibetana* sister to a clade of *C. monbeigiana*, and *C. mollicoma*. The whole-genome consensus sequences were used for bootstrap calculation, which shows a score of 100 main branch (Supplementary Figure 4). This phylogenetic result is also consistent with their distributions, as *C. tibetana* is only distributed in QTP, whereas *C. monbeigiana* and *C. mollicoma* are distributed mainly in the Yunnan–Guizhou Plateau with some sympatric populations. The following PCA and population structure analysis also supported our phylogenetic results (Figures 1C,D). The PC1 (representing 29.4% of the total variation) distinguished *C. tibetana* from *C. monbeigiana* and *C. mollicoma* and PC2 (representing 13.0% of the total variant) distinguished between *C. monbeigiana* and *C. mollicoma*. The population structure analysis further revealed a suggested admixture between *C. monbeigiana* and *C. mollicoma*, with no signals between *C. tibetana* and the other two species.

We also found a small relative distance within *C. tibetana* individuals compared with the other two species, as evident by a shorter branch length in the NJ tree and a closer distance in PCA (Figures 1B,C). This is also reflected by a significantly lower genetic diversity (π) and average individual heterozygosity values in *C. tibetana* (0.0016/0.0136) than that in *C. monbeigiana* (0.0057/0.0443) and *C. mollicoma* (0.0030/0.0221) (Supplementary Figures 5, 6). The population differentiation between each two species pair was further estimated, and the average *F*_ST_ between *C. monbeigiana* and *C. mollicoma* was 0.109, which was significantly smaller than that between *C. tibetana*, and *C. monbeigiana*, or *C. mollicoma* (Supplementary Figure 7). In addition, we also inferred the Tajima’s D values for each species, and a distribution that shifted toward positive was detected within *C. tibetana* (Supplementary Figure 8), which indicated a population contraction and consistent with the low genetic diversity detected in this species. By contrast, a slightly positive and negative Tajima’s D was detected within *C. monbeigiana* and *C. mollicoma*, respectively (Supplementary Figure 8).

### Demographic History

We firstly used the PSMC method with five individuals per species to examine changes in effective population size (*Ne*) of each species (Supplementary Figure 9). *C. tibetana* and *C. mollicoma* both showed a peak at approximately 800 kya followed by a population shrink, which was associated with the Naynayxungla Glaciation (780–500 kya). Subsequently, *C. mollicoma* recovered its population size at approximately 100 kya but shrank again at approximately 20 kya during the last glacial maximum. The *Ne* of *C. tibetana* showed a continuous decline and nearly reached 0 in the recent 10 kya. As for *C. monbeigiana*, a huge spike was detected at ∼1.3 Mya, and all five *C. monbeigiana* individuals showed the same population history trajectory.

Although the PSMC method could not explore the gene flow between each species and only gives accurate estimation for a short time scale (only results ≥10 kya are accurate) (Patton et al., 2019), we further selected the SFS approach implemented in fastsimcoal2 to simulate the demographic fluctuations and speciation history. Due to the very small *Ne* size within *C. tibetana*, we detected a nearly flat distribution of the SFS (Supplementary Figure 10), which made the demographic inference accuracy very difficult. Furthermore, *C. tibetana* did not show any significant gene flow signals with *C. monbeigiana* or *C. mollicoma* (Supplementary Figure 11), which indicated a low influence for interpreting the speciation history between *C. monbeigiana* and *C. mollicoma*. Therefore, *C. tibetana* was not considered in the following speciation analysis.

**FIGURE 2:**
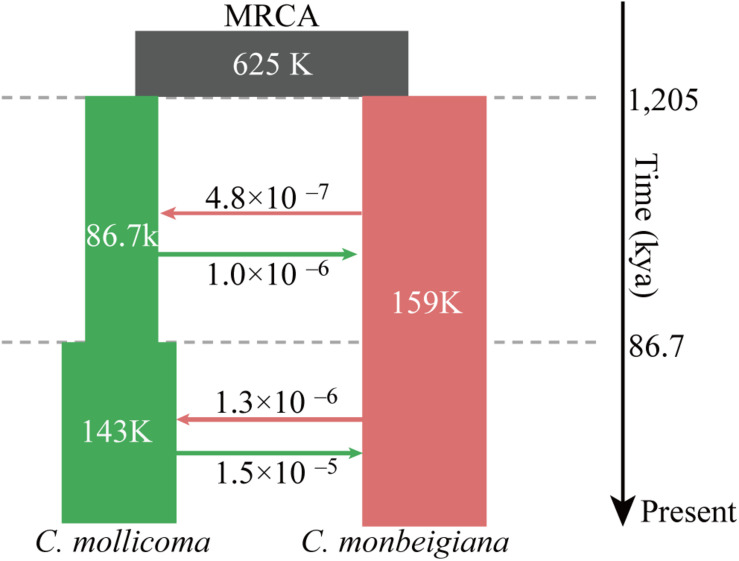
Schematic of demographic scenario modeled using fastsimcoal2. Split times (kya), population size, and migration rates corresponding to 95% CIs obtained from this model are shown in Supplementary Table 3. Estimates of gene flow between populations are given as the migration fraction per generation. The top black square was the most recent common ancestor (MRCA) to *C. monbeigiana* and *C. mollicoma*.

We tested three models (i) without gene flow, (ii) with one-way gene flow, or (iii) with two-way gene flow during the speciation events of *C. monbeigiana* and *C. mollicoma*. Bidirectional gene flow was found to be the best model. This is similar to other studies where speciation with bidirectional gene flow was common, especially in the differentiation of closely related species with no strict isolation (Han et al., 2017; Leaché et al., 2019). We then added a maximum of one instantaneous population size changes per species with the bidirectional gene flow, and the results showed the model with one population change event in *C. mollicoma* as the best model (Figure 2 and Supplementary Table 2). Within this best scenario, the speciation event between *C. mollicoma* and *C. monbeigiana* occurred at 1.20 Mya (95% confidence interval: 0.85–1.56 Mya, Figure 2 and Supplementary Table 3), in the Early Pleistocene during the Xixiabangma Glaciation (1.17–0.80 Mya) (Yu et al., 2013). *C. mollicoma* started to expand at ∼86.7 kya, which is consistent with the time estimated using PSMC. A continuous asymmetrical bidirectional gene flows was detected and was 2–11 times higher from *C. mollicoma* to *C. monbeigiana* than the reverse.

### Genomic Divergence and Selection

**FIGURE 3:**
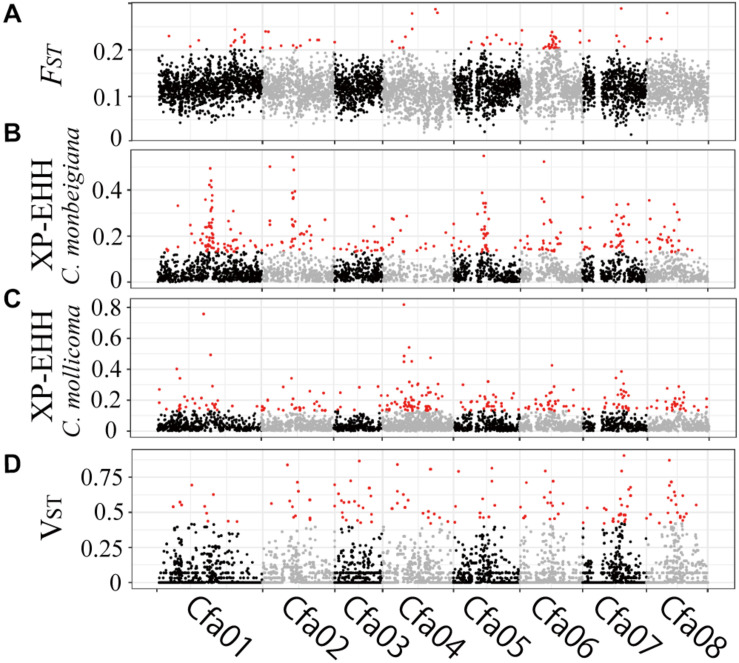
Genomic regions with high divergence or selected signals. **(A)** Pairwise genetic divergence of SNPs (*F*_ST_) in 50-kb sliding windows across all chromosomes for all comparisons. Divergence outliers (*P* < 0.01) are shown in red. Selection of SNPs (XP-EHH) in 50-kb sliding windows across all chromosomes for *C. monbeigiana* **(B)** and *C. mollicoma* **(C)**. Divergence outliers (*P* < 0.01) are shown in red. **(D)** Pairwise genetic divergence of CNVs (*V*_ST_) in 10-kb sliding windows across all chromosomes for all comparisons. Divergence outliers (*P* < 0.05) are shown in red.

To investigate the patterns of interspecific genetic differentiation across the genome, we calculated the standard measure of genetic divergence (*F*_ST_) between *C. monbeigiana* and *C. mollicoma*. A total of 252 50-kb windows (containing 291 genes) were identified as significant regions of divergency (*P* < 0.01, Figure 3A), and these genes were mainly involved in photosynthesis-related functions (e.g., carbon fixation, photosystem II repair, and photosynthetic electron transport in photosystem II) (*P* < 0.05, Supplementary Figure 12). We also identified a long region (∼4 Mb, containing 46 genes) located at chromosome 6 with a significantly high *F*_ST_ (Figure 3A). Among these genes, the *Cfa015116* gene is located at the center of this region and has a clear differentiation between *C. monbeigiana* and *C. mollicoma* (Supplementary Figure S13). The homolog of *Cfa015116* in *Arabidopsis* is *AT5G62230.2*, which encodes a receptor-like kinase, together with ER and ERL2, and governs the initial fate of protodermal cells (Shpak, 2013). The loss-of-function mutation exhibits shortened stems in *Arabidopsis* (Ikematsu et al., 2017). The divergence of *Cfa015116* may regulate the development of organs and plant height in *C. monbeigiana* and *C. mollicoma*.

Based on XP-EHH, we found 95 and 75, 50-Kb windows with significantly positive and negative XP-EHH values, containing 166 and 103 genes, respectively, suggesting that the genes have undergone selection in *C. monbeigiana* and *C. mollicoma* (Figures 3B,C). Compared with *C. mollicoma*, *C. monbeigiana* has more regions of potential positive selection. *C. monbeigiana* also has a larger number of strongly selected regions (Figures 3B,C, χ^2^ test *P* = 0.033). The results of functional enrichment analyses show that the genes related to DNA topological change are significantly enriched (*P* < 0.01) and the genes with functions in energy acquisition also tend to be enriched (cellular carbohydrate biosynthetic process, *P* < 0.01, photosynthesis, *P* = 0.0516; Supplementary Table 4). In contrast, *C. mollicoma* was mainly enriched for genes concerned with plant development (Supplementary Table 4). The enriched region of chromosome two in *C. monbeigiana* (Figure 3B) is homologous with the *Arabidopsis* locus *AT5G09810.1*, which influences germination and root growth. Furthermore, we found that 66 windows were significantly divergent and were significantly selected in a total of 6,616 windows, which occupied 20.0% (66/332) and 17.5% (66/378) of significantly inter-lineage divergence and positive selection windows, respectively. The correlation between overlapped and background windows was significant (Yates’ Correcting *t*-test, *P* < 10^–20^).

### Differentiation of Genome-Wide Copy Number Variant

To explore genomic structural change, we analyzed genomic CNVs. Using whole-genome copy number estimates from Control-FREEC (Boeva et al., 2012; Rinker et al., 2019), we identified an average of 1,168 and 1,237 CNVs, accounting for 100.1 and 84.0 Mb, in *C. monbeigiana* and *C. mollicoma*, respectively, which indicated a shorter average length of CNVs in *C. mollicoma* than that in *C. monbeigiana*. By converting to CNV genes, we observed a mean of 542 and 852 duplicated genes and 5,500 and 5,313 absent genes in the *C. monbeigiana* and *C. mollicoma* populations, respectively. The proportions of CNV were consistent with those observed in an earlier oilseed rape study (Hua et al., 2018). With respect to the differentiation between *C. monbeigiana* and *C. mollicoma*, we identified a total of 223 candidate genes with *V*_ST_ > 0.42, indicating *P-*values less than 0.005 (see section “Material and Methods”, Figure 3D). Of these 223 genes, 43 and 119 genes were gained within *C. monbeigiana* and *C. mollicoma*, respectively. The gene function analysis suggested that the set of expanded genes in *C. monbeigiana* was enriched for nutrition acquisition and biosynthesis (cellular response to phosphate starvation, *de novo* pyrimidine nucleobase biosynthetic process, photosynthesis and light harvesting, *P* < 0.05, Supplementary Table 5), whereas those in *C. mollicoma* were enriched mainly for cell division and defense response functions (*P* < 0.01, Supplementary Table 5).

## Discussion

The whole-genome analysis of *C. tibetana* revealed that the species had undergone a continuous population decline for the last 800 k years until recently (Supplementary Figure 12), indicating an extended bottleneck. This population decline also showed consistent result and suggested that *C. tibetana* has a low population genetic diversity (Supplementary Figures 5 and 9) and low individual heterozygosity (Supplementary Figure 7). This also consistent with the narrower distribution range of *C. tibetana*, as currently, it is only found along the Yarlung Zangbo River directly to the south of Medog, China (Lu et al., 2018). Because it was only discovered 2 years ago, the International Union for Conservation of Nature red list has not recorded *C. tibetana* as an endangered plant. Considering the low effective population size, low genetic variation between its individual and narrow distribution habitat, *C. tibetana* might be under high extinction risk. Thus, we recommend that the International Union for Conservation of Nature should reassess its vulnerability soon and prioritize it to ensure its long-term survival. The phylogeny, PCA, population structure, and population divergence analysis also confirm that *C. tibetana* is more different than the other two hornbeam species (Figure 1D and Supplementary Figure 8). The ABBA–BABA results (Supplementary Figure 11) further show insignificant gene flow between *C. tibetana* and *C. monbeigiana* or *C. mollicoma*, which also supports by the non-overlapped habitat range between *C. tibetana* and the other two species. Our inferred genetic relationships are different from those estimated using ITS (Lu et al., 2018). ITS sequences are usually affected by undetected paralogy, incomplete lineage sorting, and introgressive hybridization (Vilas et al., 2005; Locke et al., 2010a,b). However, our results are based on whole-genome data with population-level analysis, which likely gives more accurate relationships among the three species.

We also comprehensively assessed the speciation history of *C. monbeigiana* and *C. mollicoma*. Continuous bidirectional gene flow was found to have occurred during the speciation process. More substantial introgression from *C. mollicoma* to *C. monbeigiana* was detected than from *C. monbeigiana* to *C. mollicoma*, which might explain the higher genetic diversity within *C. monbeigiana*. Interestingly, the gene flow was found to be 10 times stronger even long after speciation, which might indicate a secondary contact between *C. mollicoma* and *C. monbeigiana* at ∼86.7 kya during the last glacial maximum. During that time, gene flow from *C. mollicoma* to *C. monbeigiana* may have been influenced by climate change, which created a condition of novel contact. We also found that the genetic divergence (*F*_ST_) between the two species was at a lower level (∼0.109) when compared with the recent studies on poplar tree (Chhatre et al., 2018; Cai et al., 2020). This may be caused by the relative gene flow between them that eliminates divergence at non-selecting regions (Han et al., 2017). Continuously high asymmetric gene flow may also explain the spike within *C. monbeigiana* in the PSMC analysis. Because the SMC family of models assume no migration and no selection, both factors could affect the *Ne* trajectory. We also performed the PSMC analysis by removing the high diverged and selected regions within *C. monbeigiana*, including the high *F*_ST_ region (*P* < 0.05, see section “Material and Methods”) between *C. monbeigiana* and *C. mollicoma* and the XP-EHH identified selected regions within *C. monbeigiana*. All the PSMC results were similar, so we could consider selection was not the main reason that caused the striking population expansion within *C. monbeigiana*. The rapid population expansion may cause by gene flow.

Selection in a different environment is a strong force for speciation, especially for the closely related species that has no strict isolation (Bertolini et al., 2018; Wang et al., 2018). Genes identified within highly diverged and selected regions were mainly involved in plant development, energy storage and consumption, and nutrition acquisition, which were mainly consistent with their habitats. For example, *C. mollicoma* prefers stony hillsides at low altitudes and is short, whereas *C. monbeigiana* prefers barren soil at high altitudes and is tall. We also identified a total of 66 windows where both species are under positive selection and exhibit high inter-lineage divergence. Most (60/66) of the overlapping genomic regions were present in *C. monbeigiana*, which also shows enrichment of genes involved in energy transfer and acquisition (Supplementary Table 6). Only six windows containing 11 genes were identified in *C. mollicoma*. Among these genes, two (*Cfa006227* and *Cfa010395* homologs to *BGLU42* and *CYP82C4* in *Arabidopsis*, respectively) are associated with root iron deficiency response (Zamioudis et al., 2014; Rajniak et al., 2018), and one (*Cfa012602* homolog to *BOB1* in *Arabidopsis*) might affect plant thermotolerance (Perez et al., 2009). *CYP82C4* (homolog to *Cfa010395*) encodes a cytochrome P450 enzyme that is involved in the early iron (Fe) deficiency response in *A. thaliana* through a FIT-dependent pathway, which could regulate the catecholic coumarins exudates in the rhizosphere in response to an iron deficiency under acidic conditions (Murgia et al., 2011; Rajniak et al., 2018). *BGLU42* (homolog to *Cfa006227*) encodes a β-glucosidase as a novel component of the induced systemic resistance signaling pathway, which might influence the nutrient in rhizosphere. In addition, *BGLU42* is also associated with plant survival under iron deficiency. *BOB1* (homolog to *Cfa012602*) encodes a non-canonical small heat shock protein required for both development and thermotolerance. Loss-of-function mutants are embryo lethal, and a partial loss-of-function allele decreases the plant thermotolerance and exhibits pleiotropic developmental phenotypes (Kaplinsky, 2009; Perez et al., 2009). These reproductive proteins have diverged rapidly across lineages and emerged as candidates involved in the plant adaptation to the diverse environment.

Copy number variant has been reported to be associated with the evolution in *Drosophila melanogaster* (Schrider et al., 2016), *Oryza* (Bai et al., 2016), and with adaptation in humans (Iskow et al., 2012; Hsieh et al., 2019). In our study, we found that the 2 (*Cfa002178* and *Cfa014950*) of 10 genes associated with DNA topology change were selected within the *C. monbeigiana* linkage. *Cfa002178* homolog to TOP3α in *Arabidopsis*, which encodes topoisomerase 3α, involved in the suppression of crossover recombination in somatic cells and DNA repair in both mammals and *A. thaliana* (Hartung et al., 2008; Knoll and Puchta, 2011). Further analysis of selected CNVs showed enrichment of functions that are similar to the SNP-based analysis.

In conclusion, our study illustrated the occurrence of bidirectional gene flow during the speciation between *C. monbeigiana* and *C. monbeigiana* and a consistent function between the diverged and selected genes in both the dataset of SNPs and CNVs. Furthermore, we reported a high extinction risk for *C. tibetana*, which has a small population size and exhibits low genetic diversity. Our study could enrich our understanding of the gene flow and natural selection during the speciation process and also serve as a good methodological reference for future research.

## Data Availability Statement

The datasets presented in this study can be found in online repositories. The name of the repository and accession number can be found here: https://www.ncbi.nlm.nih.gov/, PRJNA608439.

## Author Contributions

YY designed the experiments and coordinated the project. ZZ, YL, and ZL collected all the samples. YL, XD, HH, and MY performed the DNA extraction. ZZ, ML, and GL performed the raw sequencing data filtering, mapping, and SNP calling. ZZ and YL performed the population analysis and wrote the raw manuscript. YY reproved the manuscript. NS was a native speaker and polished the English writing. XZ help to polished the English writing. All authors read and approved the final manuscript.

## Conflict of Interest

The authors declare that the research was conducted in the absence of any commercial or financial relationships that could be construed as a potential conflict of interest.

## Footnotes

1 https://github.com/vsbuffalo/scythe

2 https://github.com/najoshi/sickle

3 https://github.com/rambaut/figtree
