# Supplementary material for: Whole-Genome Diversification Analysis of the Hornbeam Species Reveals Speciation and Adaptation Among Closely Related Species
Source: Front Plant Sci. 2021 Feb 10;12:581704. doi: 10.3389/fpls.2021.581704 (PMC7902934; doi:10.3389/fpls.2021.581704)
Supplement: Supplementary file 1 [file Data_Sheet_1.docx]

**SUPPLEMENTARY DATA**

**Whole genome diversification analysis of the hornbeam species reveals speciation and adaptation among closely related species**

Zheng and Li et al.

# Supplementary Tables:

## Table S1. Overview of sample information and sequencing statistics.

| **Sample ID** | **Species** | **Country/Region** | **Clean Reads (Gbp)** | **Genome Coverage (%)** | **Effective Depth** |
| --- | --- | --- | --- | --- | --- |
| CM02 | *C. monbeigiana* | Kunming, China | 11.60 | 0.82 | 22.36 |
| CM03 | *C. monbeigiana* | Kunming, China | 10.33 | 0.82 | 20.33 |
| CM04 | *C. monbeigiana* | Yunnan, China | 9.65 | 0.81 | 19.89 |
| CM05 | *C. monbeigiana* | Yunnan, China | 9.82 | 0.82 | 19.75 |
| CM06 | *C. monbeigiana* | Yunnan, China | 11.88 | 0.82 | 23.56 |
| CM07 | *C. monbeigiana* | Yunnan, China | 11.69 | 0.83 | 23.40 |
| CM08 | *C. monbeigiana* | Yunnan, China | 13.22 | 0.84 | 26.52 |
| CM09 | *C. monbeigiana* | Yunnan, China | 9.57 | 0.81 | 16.79 |
| CM10 | *C. monbeigiana* | Yunnan, China | 8.57 | 0.81 | 15.92 |
| CM11 | *C. monbeigiana* | Yunnan, China | 10.63 | 0.82 | 18.85 |
| CM12 | *C. monbeigiana* | Kunming, China | 9.47 | 0.81 | 19.06 |
| CM14 | *C. monbeigiana* | Kunming, China | 28.80 | 0.86 | 54.54 |
| CM16 | *C. monbeigiana* | Yunnan, China | 7.87 | 0.82 | 16.53 |
| CRM02 | *C. mollicoma* | Yunnan, China | 9.22 | 0.80 | 18.84 |
| CRM03 | *C. mollicoma* | Guizhou, China | 9.37 | 0.79 | 17.45 |
| CRM04 | *C. mollicoma* | Guizhou, China | 9.68 | 0.78 | 17.33 |
| CRM05 | *C. mollicoma* | Guizhou, China | 7.92 | 0.77 | 12.84 |
| CRM06 | *C. mollicoma* | Yunnan, China | 8.94 | 0.80 | 17.18 |
| CRM07 | *C. mollicoma* | Guizhou, China | 7.74 | 0.77 | 13.10 |
| CRM08 | *C. mollicoma* | Yunnan, China | 8.42 | 0.80 | 17.57 |
| CRM10 | *C. mollicoma* | Guizhou, China | 8.15 | 0.77 | 12.51 |
| CRM12 | *C. mollicoma* | Guizhou, China | 11.69 | 0.77 | 11.24 |
| CT01 | *C. tibetana* | Xizang, China | 10.58 | 0.76 | 22.80 |
| CT02 | *C. tibetana* | Xizang, China | 11.00 | 0.76 | 23.85 |
| CT03 | *C. tibetana* | Xizang, China | 9.45 | 0.75 | 17.92 |
| CT05 | *C. tibetana* | Xizang, China | 10.05 | 0.75 | 19.33 |
| CT06 | *C. tibetana* | Xizang, China | 10.86 | 0.76 | 20.74 |
| CT07 | *C. tibetana* | Xizang, China | 13.90 | 0.77 | 25.99 |
| CT08 | *C. tibetana* | Xizang, China | 13.20 | 0.77 | 24.68 |
| CT09 | *C. tibetana* | Xizang, China | 10.63 | 0.76 | 20.12 |
| CT10 | *C. tibetana* | Xizang, China | 8.88 | 0.74 | 19.37 |
| CT11 | *C. tibetana* | Xizang, China | 9.75 | 0.75 | 18.59 |
| CT12 | *C. tibetana* | Xizang, China | 10.02 | 0.75 | 19.57 |
| CT13 | *C. tibetana* | Xizang, China | 11.55 | 0.76 | 25.39 |
| CT14 | *C. tibetana* | Xizang, China | 11.17 | 0.76 | 24.39 |
| CT15 | *C. tibetana* | Xizang, China | 9.59 | 0.76 | 20.67 |
| CT16 | *C. tibetana* | Xizang, China | 11.62 | 0.76 | 22.66 |
| CT17 | *C. tibetana* | Xizang, China | 11.01 | 0.76 | 21.47 |
| CT18 | *C. tibetana* | Xizang, China | 9.94 | 0.75 | 19.41 |
| CT19 | *C. tibetana* | Xizang, China | 7.71 | 0.75 | 15.49 |
| CT20 | *C. tibetana* | Xizang, China | 8.69 | 0.75 | 17.14 |
| CT21 | *C. tibetana* | Xizang, China | 8.89 | 0.75 | 19.19 |
| CT23 | *C. tibetana* | Xizang, China | 9.21 | 0.74 | 18.13 |
| CT24 | *C. tibetana* | Xizang, China | 9.45 | 0.76 | 20.48 |
| CT25 | *C. tibetana* | Xizang, China | 8.91 | 0.76 | 18.49 |
| CT26 | *C. tibetana* | Xizang, China | 8.88 | 0.75 | 19.22 |
| CT27 | *C. tibetana* | Xizang, China | 11.31 | 0.77 | 24.46 |
| CT28 | *C. tibetana* | Xizang, China | 9.38 | 0.75 | 19.90 |
| CT29 | *C. tibetana* | Xizang, China | 9.44 | 0.76 | 20.60 |
| CT30 | *C. tibetana* | Xizang, China | 8.27 | 0.75 | 17.30 |
| CT31 | *C. tibetana* | Xizang, China | 15.50 | 0.79 | 31.75 |
| CT32 | *C. tibetana* | Xizang, China | 8.20 | 0.77 | 17.35 |
| CT33 | *C. tibetana* | Xizang, China | 9.14 | 0.75 | 13.84 |
| Ccor01 | *C. cordata* | Honshu, Japan | 27.24 | 0.87 | 49.98 |
| Otr05 | *Ostrya trichocarpa* | Guizhou, China | 9.02 | 0.74 | 15.02 |

## Table S2. Likelihood analysis of all demographic history models.

|  | **Negative log likelihood (Lhood)** | **No. of parameters (d)** | **AIC*** | **ΔAIC†** |
| --- | --- | --- | --- | --- |
| **Model1** | 19,563,774 | 4 | 90,094,518 | 769,258 |
| **Model2** | 19,481,501 | 6 | 89,715,637 | 243,230 |
| **Model3** | 19,564,331 | 5 | 90,097,084 | 624,677 |
| **Model4** | 19,565,242 | 5 | 90,101,279 | 628,872 |
| **Model5** | 19,428,682 | 10 | 89,472,407 | 0 |
| **Model6** | 19,463,552 | 10 | 89,632,987 | 160,580 |
| **Model7** | 19,464,071 | 14 | 89,635,386 | 162,979 |
| **Model8** | 19,433,059 | 14 | 89,492,572 | 20,165 |
| **Model9** | 19,838,232 | 6 | 91,358,446 | 1,886,039 |
| ***AIC=2d-2ln(Lhood)** | |  |  |  |
| **†ΔAIC=AIC-min(AIC)** | |  |  |  |

## Table S3. Inferred parameters estimated with 95% confidence intervals for the best-fitting demographic scenario modelled in fastsimcoal2 which is shown in Figure 2. Estimates of gene flow between populations are given as the migration fraction per generation.

| **Parameters** | **Meaning** | **Point estimation** | **95% confidence intervals** | |
| --- | --- | --- | --- | --- |
|  |  |  | **Lower bound** | **Upper bound** |
| ANCSIZE | Ancient population size | 50,641 | 46,313 | 54,969 |
| MIG12 | Migration from *C. mollicoma* to *C. monbeigiana* | 6.08E-06 | 6.07E-06 | 6.09E-06 |
| MIG21 | Migration from *C. monbeigiana* to *C. mollicoma* | 2.35E-06 | 2.34E-06 | 2.36E-06 |
| NPOP1 | *C. mollicoma* population size now | 1.14E+05 | 1.13E+05 | 1.14E+05 |
| NPOP2 | *C. monbeigiana* population size now | 2.50E+05 | 2.49E+05 | 2.50E+05 |
| RESIZE | Fold of population size change at division | 0.20 | 0.19 | 0.22 |
| TDIV | Division time | 5.02E+06 | 4.59E+06 | 5.46E+06 |

## Table S4. Results of Gene Ontology enrichment analysis of positively selected genes (PSGs) using SNPs.

| **GO ID** | **GO name** | **Ontology** | **PSGs** | **Annotated** | ***P*** |
| --- | --- | --- | --- | --- | --- |
| *C. monbeigiana* | | | | | |
| GO:0008152 | metabolic process | BP | 44 | 7021 | 0.0003 |
| GO:0006265 | DNA topological change | BP | 2 | 10 | 0.0010 |
| GO:0006414 | translational elongation | BP | 2 | 22 | 0.0051 |
| GO:0034637 | cellular carbohydrate biosynthetic process | BP | 3 | 90 | 0.0095 |
| GO:0051315 | attachment of mitotic spindle microtubules to kinetochore | BP | 1 | 2 | 0.0097 |
| GO:0007080 | mitotic metaphase plate congression | BP | 1 | 2 | 0.0097 |
| GO:0051310 | metaphase plate congression | BP | 1 | 2 | 0.0097 |
| GO:0071103 | DNA conformation change | BP | 2 | 32 | 0.0105 |
| GO:0016051 | carbohydrate biosynthetic process | BP | 3 | 105 | 0.0144 |
| GO:0017183 | peptidyl-diphthamide biosynthetic process from peptidyl-histidine | BP | 1 | 3 | 0.0146 |
| GO:0051560 | mitochondrial calcium ion homeostasis | BP | 1 | 3 | 0.0146 |
| GO:0002182 | cytoplasmic translational elongation | BP | 1 | 3 | 0.0146 |
| GO:0017182 | peptidyl-diphthamide metabolic process | BP | 1 | 3 | 0.0146 |
| GO:0018202 | peptidyl-histidine modification | BP | 1 | 3 | 0.0146 |
| GO:0050000 | chromosome localization | BP | 1 | 3 | 0.0146 |
| GO:0051303 | establishment of chromosome localization | BP | 1 | 3 | 0.0146 |
| GO:1900247 | regulation of cytoplasmic translational elongation | BP | 1 | 3 | 0.0146 |
| GO:2000765 | regulation of cytoplasmic translation | BP | 1 | 3 | 0.0146 |
| GO:0016579 | protein deubiquitination | BP | 2 | 39 | 0.0154 |
| GO:0055114 | oxidation-reduction process | BP | 13 | 1431 | 0.0167 |
| GO:0070646 | protein modification by small protein removal | BP | 2 | 43 | 0.0185 |
| GO:0002181 | cytoplasmic translation | BP | 1 | 4 | 0.0194 |
| GO:0044262 | cellular carbohydrate metabolic process | BP | 3 | 133 | 0.0268 |
| GO:0051274 | beta-glucan biosynthetic process | BP | 2 | 54 | 0.0284 |
| GO:0006448 | regulation of translational elongation | BP | 1 | 6 | 0.0289 |
| GO:0006874 | cellular calcium ion homeostasis | BP | 1 | 6 | 0.0289 |
| GO:0055074 | calcium ion homeostasis | BP | 1 | 6 | 0.0289 |
| GO:0072503 | cellular divalent inorganic cation homeostasis | BP | 1 | 6 | 0.0289 |
| GO:0072507 | divalent inorganic cation homeostasis | BP | 1 | 6 | 0.0289 |
| GO:0051273 | beta-glucan metabolic process | BP | 2 | 55 | 0.0294 |
| GO:0034645 | cellular macromolecule biosynthetic process | BP | 14 | 1721 | 0.0312 |
| GO:0009059 | macromolecule biosynthetic process | BP | 14 | 1724 | 0.0317 |
| GO:0008608 | attachment of spindle microtubules to kinetochore | BP | 1 | 7 | 0.0337 |
| GO:0009250 | glucan biosynthetic process | BP | 2 | 63 | 0.0377 |
| GO:0051276 | chromosome organization | BP | 3 | 164 | 0.0455 |
| GO:0033692 | cellular polysaccharide biosynthetic process | BP | 2 | 71 | 0.0468 |
| GO:0006074 | (1->3)-beta-D-glucan metabolic process | BP | 1 | 10 | 0.0478 |
| GO:0006075 | (1->3)-beta-D-glucan biosynthetic process | BP | 1 | 10 | 0.0478 |
| GO:0006417 | regulation of translation | BP | 1 | 10 | 0.0478 |
| GO:0034248 | regulation of cellular amide metabolic process | BP | 1 | 10 | 0.0478 |
| GO:0000271 | polysaccharide biosynthetic process | BP | 2 | 72 | 0.0480 |
| GO:0008083 | growth factor activity | MF | 2 | 7 | 0.0006 |
| GO:0030545 | receptor regulator activity | MF | 2 | 8 | 0.0008 |
| GO:0048018 | receptor ligand activity | MF | 2 | 8 | 0.0008 |
| GO:0003916 | DNA topoisomerase activity | MF | 2 | 10 | 0.0013 |
| GO:0005102 | signaling receptor binding | MF | 2 | 14 | 0.0025 |
| GO:0004609 | phosphatidylserine decarboxylase activity | MF | 1 | 3 | 0.0162 |
| GO:0003918 | DNA topoisomerase type II (double strand cut, ATP-hydrolyzing) activity | MF | 1 | 4 | 0.0215 |
| GO:0061505 | DNA topoisomerase II activity | MF | 1 | 4 | 0.0215 |
| GO:0036459 | thiol-dependent ubiquitinyl hydrolase activity | MF | 2 | 43 | 0.0227 |
| GO:0019783 | ubiquitin-like protein-specific protease activity | MF | 2 | 43 | 0.0227 |
| GO:0101005 | ubiquitinyl hydrolase activity | MF | 2 | 45 | 0.0247 |
| GO:0016491 | oxidoreductase activity | MF | 15 | 1632 | 0.0271 |
| GO:0004040 | amidase activity | MF | 1 | 6 | 0.0321 |
| GO:0004576 | oligosaccharyl transferase activity | MF | 1 | 6 | 0.0321 |
| GO:0003917 | DNA topoisomerase type I activity | MF | 1 | 6 | 0.0321 |
| GO:0004124 | cysteine synthase activity | MF | 1 | 7 | 0.0373 |
| *C. mollicoma* | | | | | |
| GO:0010088 | phloem development | BP | 4 | 24 | 0.0000 |
| GO:0010087 | phloem or xylem histogenesis | BP | 4 | 29 | 0.0000 |
| GO:0009888 | tissue development | BP | 4 | 41 | 0.0000 |
| GO:0048856 | anatomical structure development | BP | 5 | 127 | 0.0000 |
| GO:0032502 | developmental process | BP | 5 | 139 | 0.0000 |
| GO:0060918 | auxin transport | BP | 1 | 4 | 0.0079 |
| GO:0009914 | hormone transport | BP | 1 | 4 | 0.0079 |
| GO:0051225 | spindle assembly | BP | 1 | 7 | 0.0137 |
| GO:0007051 | spindle organization | BP | 1 | 12 | 0.0234 |
| GO:0010817 | regulation of hormone levels | BP | 1 | 15 | 0.0292 |
| GO:0070925 | organelle assembly | BP | 1 | 20 | 0.0388 |
| GO:0099402 | plant organ development | BP | 1 | 24 | 0.0463 |
| GO:0043531 | ADP binding | MF | 3 | 63 | 0.0004 |
| GO:0010333 | terpene synthase activity | MF | 2 | 35 | 0.0028 |
| GO:0016838 | carbon-oxygen lyase activity, acting on phosphates | MF | 2 | 38 | 0.0032 |
| GO:0004222 | metalloendopeptidase activity | MF | 2 | 63 | 0.0087 |
| GO:0008237 | metallopeptidase activity | MF | 2 | 85 | 0.0154 |
| GO:0016829 | lyase activity | MF | 3 | 255 | 0.0190 |
| GO:0016835 | carbon-oxygen lyase activity | MF | 2 | 106 | 0.0234 |
| GO:0016844 | strictosidine synthase activity | MF | 1 | 12 | 0.0266 |
| GO:0016843 | amine-lyase activity | MF | 1 | 12 | 0.0266 |
| GO:0000287 | magnesium ion binding | MF | 2 | 115 | 0.0272 |
| GO:0046872 | metal ion binding | MF | 8 | 1678 | 0.0280 |
| GO:0043169 | cation binding | MF | 8 | 1724 | 0.0323 |
| GO:0005506 | iron ion binding | MF | 3 | 350 | 0.0427 |
| GO:0016705 | oxidoreductase activity, acting on paired donors, with incorporation or reduction of molecular oxygen | MF | 3 | 365 | 0.0474 |

## Table S5. Results of Gene Ontology enrichment analysis of positively selected genes (PSGs) using CNVs.

| **GO ID** | **GO name** | **Ontology** | **PSGs** | **Annotated** | ***P*** |
| --- | --- | --- | --- | --- | --- |
| *C. monbeigiana* | | | | | |
| GO:0016036 | cellular response to phosphate starvation | BP | 1 | 4 | 0.0075 |
| GO:0046417 | chorismate metabolic process | BP | 1 | 4 | 0.0075 |
| GO:0006207 | de novo' pyrimidine nucleobase biosynthetic process | BP | 1 | 6 | 0.0112 |
| GO:0019346 | transsulfuration | BP | 1 | 6 | 0.0112 |
| GO:0009092 | homoserine metabolic process | BP | 1 | 6 | 0.0112 |
| GO:0009267 | cellular response to starvation | BP | 1 | 6 | 0.0112 |
| GO:0042594 | response to starvation | BP | 1 | 6 | 0.0112 |
| GO:0050667 | homocysteine metabolic process | BP | 1 | 6 | 0.0112 |
| GO:0009991 | response to extracellular stimulus | BP | 1 | 7 | 0.0130 |
| GO:0031667 | response to nutrient levels | BP | 1 | 7 | 0.0130 |
| GO:0031668 | cellular response to extracellular stimulus | BP | 1 | 7 | 0.0130 |
| GO:0031669 | cellular response to nutrient levels | BP | 1 | 7 | 0.0130 |
| GO:0071496 | cellular response to external stimulus | BP | 1 | 7 | 0.0130 |
| GO:0006206 | pyrimidine nucleobase metabolic process | BP | 1 | 9 | 0.0167 |
| GO:0019856 | pyrimidine nucleobase biosynthetic process | BP | 1 | 9 | 0.0167 |
| GO:0044237 | cellular metabolic process | BP | 14 | 4939 | 0.0200 |
| GO:0006032 | chitin catabolic process | BP | 1 | 12 | 0.0222 |
| GO:0046348 | amino sugar catabolic process | BP | 1 | 12 | 0.0222 |
| GO:1901072 | glucosamine-containing compound catabolic process | BP | 1 | 12 | 0.0222 |
| GO:0006801 | superoxide metabolic process | BP | 1 | 13 | 0.0240 |
| GO:0016998 | cell wall macromolecule catabolic process | BP | 1 | 13 | 0.0240 |
| GO:0006026 | aminoglycan catabolic process | BP | 1 | 13 | 0.0240 |
| GO:0006030 | chitin metabolic process | BP | 1 | 14 | 0.0259 |
| GO:0006022 | aminoglycan metabolic process | BP | 1 | 15 | 0.0277 |
| GO:1901071 | glucosamine-containing compound catabolic process | BP | 1 | 16 | 0.0295 |
| GO:0046112 | nucleobase biosynthetic process | BP | 1 | 18 | 0.0331 |
| GO:0006040 | amino sugar metabolic process | BP | 1 | 19 | 0.0349 |
| GO:0009765 | photosynthesis, light harvesting | BP | 1 | 21 | 0.0386 |
| GO:0006534 | cysteine metabolic process | BP | 1 | 21 | 0.0386 |
| GO:0009112 | nucleobase metabolic process | BP | 1 | 21 | 0.0386 |
| GO:1901136 | carbohydrate derivative catabolic process | BP | 1 | 24 | 0.0439 |
| GO:0001522 | pseudouridine synthesis | BP | 1 | 26 | 0.0475 |
| GO:0009987 | cellular process | BP | 15 | 5988 | 0.0483 |
| GO:0016866 | intramolecular transferase activity | MF | 2 | 47 | 0.0038 |
| GO:0004152 | dihydroorotate dehydrogenase activity | MF | 1 | 2 | 0.0039 |
| GO:0016635 | oxidoreductase activity, acting on the CH-CH group of donors, quinone or related compound as acceptor | MF | 1 | 2 | 0.0039 |
| GO:0043531 | ADP binding | MF | 2 | 63 | 0.0066 |
| GO:0004106 | chorismate mutase activity | MF | 1 | 4 | 0.0078 |
| GO:0004784 | superoxide dismutase activity | MF | 1 | 7 | 0.0136 |
| GO:0016721 | oxidoreductase activity, acting on superoxide radicals as acceptor | MF | 1 | 7 | 0.0136 |
| GO:0030170 | pyridoxal phosphate binding | MF | 2 | 113 | 0.0203 |
| GO:0070279 | vitamin B6 binding | MF | 2 | 113 | 0.0203 |
| GO:0004568 | chitinase activity | MF | 1 | 12 | 0.0232 |
| GO:0045300 | acyl-[acyl-carrier-protein] desaturase activity | MF | 1 | 12 | 0.0232 |
| GO:0019843 | rRNA binding | MF | 1 | 17 | 0.0327 |
| GO:0016717 | oxidoreductase activity, acting on paired donors, with oxidation of a pair of donors resulting in the reduction of molecular oxygen to two molecules of wate | MF | 1 | 17 | 0.0327 |
| GO:0019842 | vitamin binding | MF | 2 | 148 | 0.0335 |
| GO:0009982 | pseudouridine synthase activity | MF | 1 | 22 | 0.0421 |
| GO:0016853 | isomerase activity | MF | 2 | 176 | 0.0458 |
| *C. mollicoma* | | | | | |
| GO:0051301 | cell division | BP | 2 | 19 | 0.0018 |
| GO:0006952 | defense response | BP | 3 | 89 | 0.0031 |
| GO:0022900 | electron transport chain | BP | 2 | 36 | 0.0063 |
| GO:0009236 | cobalamin biosynthetic process | BP | 1 | 2 | 0.0066 |
| GO:0007059 | chromosome segregation | BP | 2 | 52 | 0.0128 |
| GO:0051225 | spindle assembly | BP | 1 | 7 | 0.0230 |
| GO:0015743 | malate transport | BP | 1 | 13 | 0.0424 |
| GO:0016844 | strictosidine synthase activity | MF | 2 | 12 | 0.0006 |
| GO:0016843 | amine-lyase activity | MF | 2 | 12 | 0.0006 |
| GO:0009055 | electron transfer activity | MF | 4 | 156 | 0.0012 |
| GO:0016840 | carbon-nitrogen lyase activity | MF | 2 | 29 | 0.0035 |
| GO:0016852 | sirohydrochlorin cobaltochelatase activity | MF | 1 | 2 | 0.0061 |
| GO:0016829 | lyase activity | MF | 4 | 255 | 0.0073 |
| GO:0004129 | cytochrome-c oxidase activity | MF | 1 | 4 | 0.0121 |
| GO:0015002 | heme-copper terminal oxidase activity | MF | 1 | 4 | 0.0121 |
| GO:0016675 | oxidoreductase activity, acting on a heme group of donors | MF | 1 | 4 | 0.0121 |
| GO:0016676 | oxidoreductase activity, acting on a heme group of donors, oxygen as acceptor | MF | 1 | 4 | 0.0121 |
| GO:0046914 | transition metal ion binding | MF | 8 | 1096 | 0.0158 |
| GO:0008017 | microtubule binding | MF | 2 | 74 | 0.0211 |
| GO:0016491 | oxidoreductase activity | MF | 10 | 1632 | 0.0216 |
| GO:0005507 | copper ion binding | MF | 2 | 87 | 0.0286 |
| GO:0015078 | proton transmembrane transporter activity | MF | 2 | 88 | 0.0292 |
| GO:0015631 | tubulin binding | MF | 2 | 88 | 0.0292 |
| GO:0008270 | zinc ion binding | MF | 5 | 613 | 0.0369 |
| GO:0004713 | protein tyrosine kinase activity | MF | 1 | 13 | 0.0387 |
| GO:0003968 | RNA-directed 5'-3' RNA polymerase activity | MF | 1 | 16 | 0.0475 |

## Table S6. Results of Gene Ontology enrichment analysis of genes under significantly selection and divergence.

| **GO ID** | **GO name** | **Ontology** | **PSGs** | **Annotated** | ***P*** |
| --- | --- | --- | --- | --- | --- |
| *C. monbeigiana* | | | | | |
| GO:0015743 | malate transport | BP | 2 | 13 | 0.0019 |
| GO:0006835 | dicarboxylic acid transport | BP | 2 | 13 | 0.0019 |
| GO:0015740 | C4-dicarboxylate transport | BP | 2 | 13 | 0.0019 |
| GO:0006414 | translational elongation | BP | 2 | 22 | 0.0055 |
| GO:0006952 | defense response | BP | 3 | 89 | 0.0103 |
| GO:0071103 | DNA conformation change | BP | 2 | 32 | 0.0114 |
| GO:0015849 | organic acid transport | BP | 2 | 39 | 0.0167 |
| GO:0046942 | carboxylic acid transport | BP | 2 | 39 | 0.0167 |
| GO:0006950 | response to stress | BP | 6 | 421 | 0.0192 |
| GO:0036297 | interstrand cross-link repair | BP | 1 | 5 | 0.0252 |
| GO:0015711 | organic anion transport | BP | 2 | 52 | 0.0286 |
| GO:0007023 | post-chaperonin tubulin folding pathway | BP | 1 | 6 | 0.0301 |
| GO:0006448 | regulation of translational elongation | BP | 1 | 6 | 0.0301 |
| GO:0008608 | attachment of spindle microtubules to kinetochore | BP | 1 | 7 | 0.0351 |
| GO:0030026 | cellular manganese ion homeostasis | BP | 1 | 7 | 0.0351 |
| GO:0042775 | mitochondrial ATP synthesis coupled electron transport | BP | 1 | 7 | 0.0351 |
| GO:0043647 | inositol phosphate metabolic process | BP | 1 | 7 | 0.0351 |
| GO:0046173 | polyol biosynthetic process | BP | 1 | 7 | 0.0351 |
| GO:0055071 | manganese ion homeostasis | BP | 1 | 7 | 0.0351 |
| GO:0016310 | phosphorylation | BP | 9 | 919 | 0.0399 |
| GO:0044260 | cellular macromolecule metabolic process | BP | 23 | 3278 | 0.0413 |
| GO:0006075 | (1->3)-beta-D-glucan biosynthetic process | BP | 1 | 10 | 0.0497 |
| GO:0006265 | DNA topological change | BP | 1 | 10 | 0.0497 |
| GO:0006074 | (1->3)-beta-D-glucan metabolic process | BP | 1 | 10 | 0.0497 |
| GO:0006417 | regulation of translation | BP | 1 | 10 | 0.0497 |
| GO:0034248 | regulation of cellular amide metabolic process | BP | 1 | 10 | 0.0497 |
| GO:0140097 | catalytic activity, acting on DNA | BP | 3 | 97 | 0.0089 |
| GO:0036094 | small molecule binding | MF | 18 | 2466 | 0.0171 |
| GO:0000166 | nucleotide binding | MF | 17 | 2289 | 0.0177 |
| GO:1901265 | nucleoside phosphate binding | MF | 17 | 2289 | 0.0177 |
| GO:0032553 | ribonucleotide binding | MF | 15 | 1970 | 0.0216 |
| GO:0050664 | oxidoreductase activity, acting on NAD(P)H, oxygen as acceptor | MF | 1 | 5 | 0.0218 |
| GO:0043167 | ion binding | MF | 25 | 3935 | 0.0234 |
| GO:0043168 | anion binding | MF | 17 | 2369 | 0.0243 |
| GO:0097367 | carbohydrate derivative binding | MF | 15 | 1998 | 0.0243 |
| GO:0003917 | DNA topoisomerase type I activity | MF | 1 | 6 | 0.0261 |
| GO:0004040 | amidase activity | MF | 1 | 6 | 0.0261 |
| GO:0008121 | ubiquinol-cytochrome-c reductase activity | MF | 1 | 6 | 0.0261 |
| GO:0016681 | oxidoreductase activity, acting on diphenols and related substances as donors, cytochrome as acceptor | MF | 1 | 6 | 0.0261 |
| GO:0016301 | kinase activity | MF | 9 | 998 | 0.0298 |
| GO:0004672 | protein kinase activity | MF | 8 | 838 | 0.0299 |
| GO:0005384 | manganese ion transmembrane transporter activity | MF | 1 | 7 | 0.0304 |
| GO:0035639 | purine ribonucleoside triphosphate bindi... | MF | 14 | 1874 | 0.0308 |
| GO:0008171 | O-methyltransferase activity | MF | 2 | 67 | 0.0352 |
| GO:0016772 | transferase activity, transferring phosphorus-containing groups | MF | 10 | 1205 | 0.0366 |
| GO:0032555 | purine ribonucleotide binding | MF | 14 | 1942 | 0.0402 |
| GO:0017076 | purine nucleotide binding | MF | 14 | 1951 | 0.0416 |
| GO:0003843 | 1,3-beta-D-glucan synthase activity | MF | 1 | 10 | 0.0432 |
| GO:0004707 | MAP kinase activity | MF | 1 | 10 | 0.0432 |
| GO:0003916 | DNA topoisomerase activity | MF | 1 | 10 | 0.0432 |
| GO:0008144 | drug binding | MF | 13 | 1796 | 0.0466 |
| *C. mollicoma* | | | | | |
| GO:0044275 | cellular carbohydrate catabolic process | BP | 1 | 5 | 0.0021 |
| GO:0000272 | polysaccharide catabolic process | BP | 1 | 14 | 0.0058 |
| GO:0009082 | branched-chain amino acid biosynthetic p... | BP | 1 | 18 | 0.0075 |
| GO:0009081 | branched-chain amino acid metabolic process | BP | 1 | 24 | 0.0099 |
| GO:0030243 | cellulose metabolic process | BP | 1 | 44 | 0.0181 |
| GO:0051273 | beta-glucan metabolic process | BP | 1 | 55 | 0.0226 |
| GO:0016052 | carbohydrate catabolic process | BP | 1 | 70 | 0.0288 |
| GO:0006073 | cellular glucan metabolic process | BP | 1 | 86 | 0.0352 |
| GO:0044042 | glucan metabolic process | BP | 1 | 86 | 0.0352 |
| GO:0044264 | cellular polysaccharide metabolic process | BP | 1 | 94 | 0.0385 |
| GO:0005976 | polysaccharide metabolic process | BP | 1 | 108 | 0.0441 |
| GO:0015926 | glucosidase activity | MF | 1 | 8 | 0.0023 |
| GO:0030234 | enzyme regulator activity | MF | 1 | 200 | 0.0565 |

# Supplementary Figures:


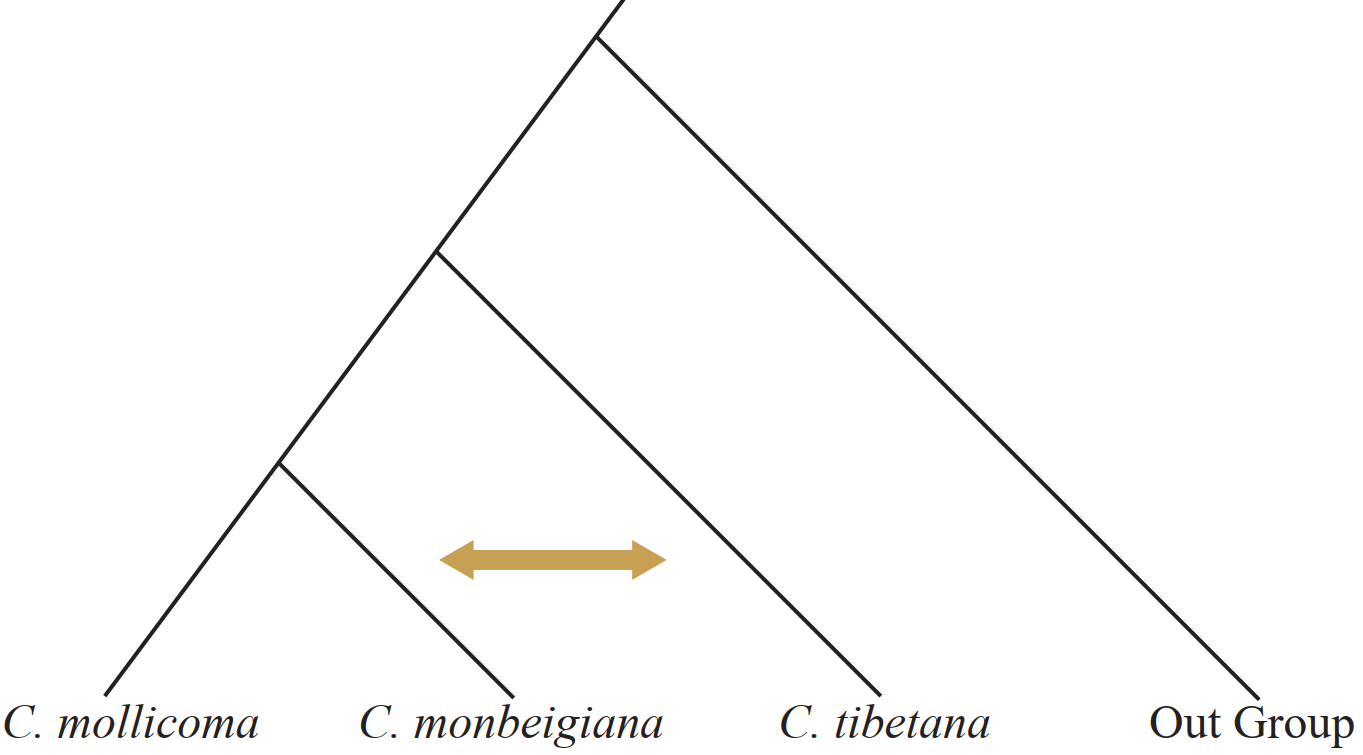


## Figure S1 ABBA-BABA model as Durand et al. derived (Durand et al., 2011).


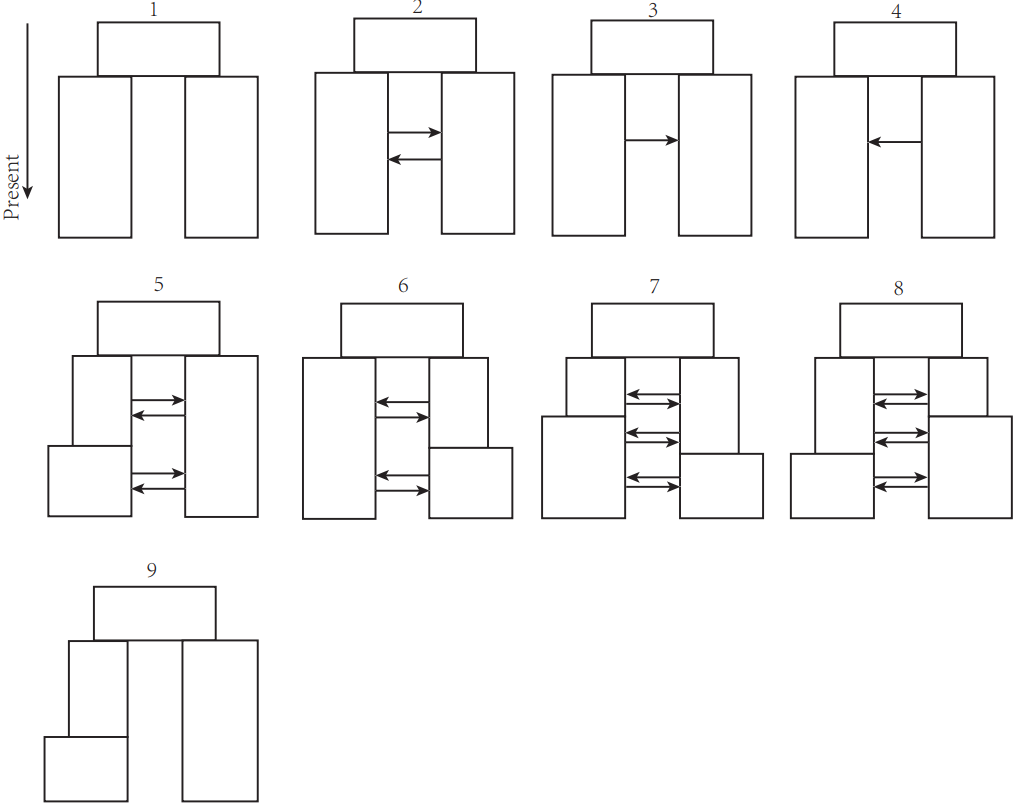


## Figure S2. Schematic diagram of different models of demographic history from ancient to recent estimated by Fastsimcoal2. The width of the box indicates population sizes of *C. mollicoma* (left) and *C. monbeigiana* (right) yaks. Arrows show the migrants between two populations.


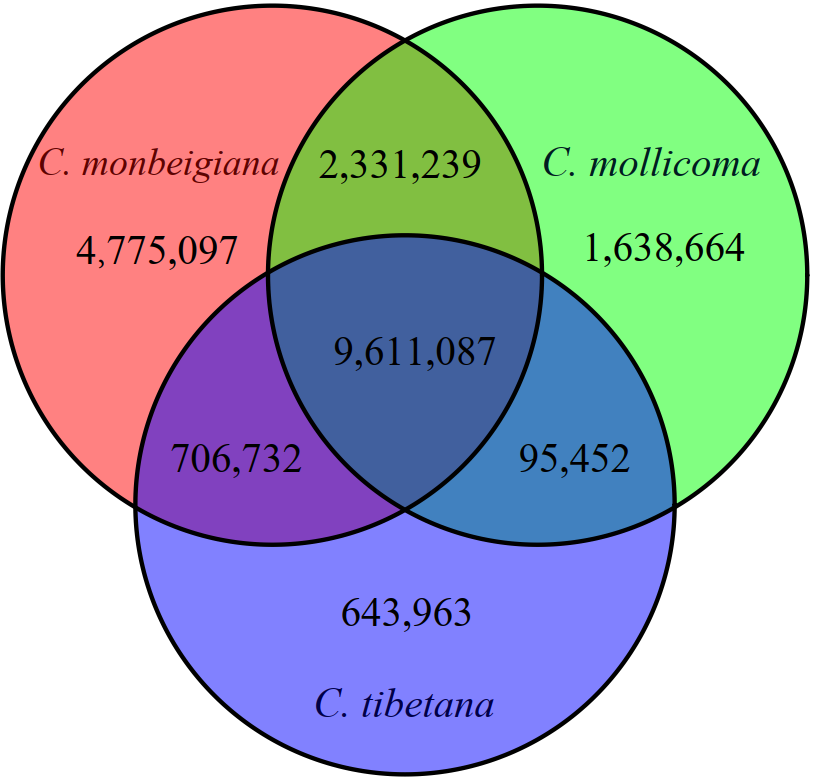


## Figure S3. Shared and unique SNPs between *C. monbeigiana*, *C. mollicoma* and *C. tibetana*.


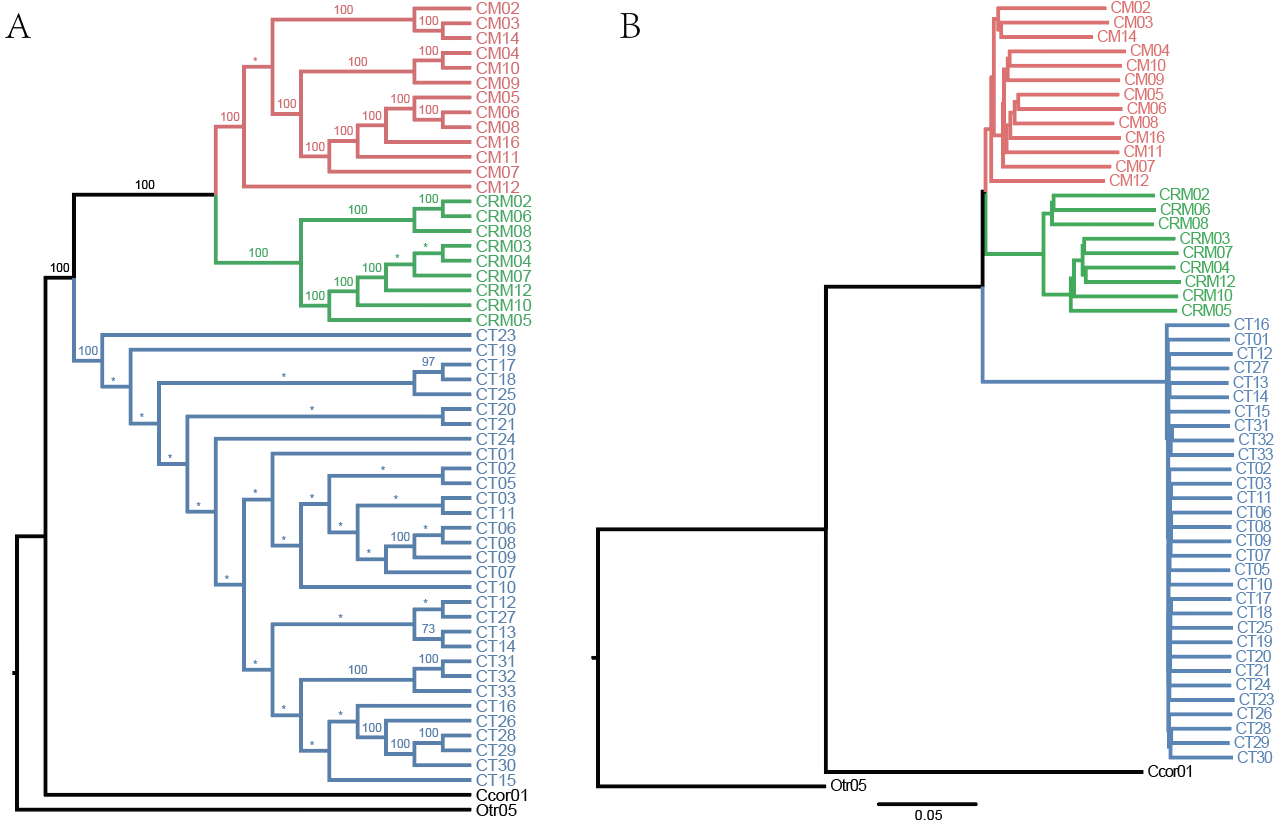


## Figure S4. Neighbor-joining (NJ) phylogenetic tree sites. (A) Neighbor-joining phylogenetic tree with branch support value for 1000 times of bootstrap by using whole-genome consensus sites. branch support lower than 70 were marked as asterisk (B) Neighbor-joining phylogenetic tree by using genome-wide SNPs.


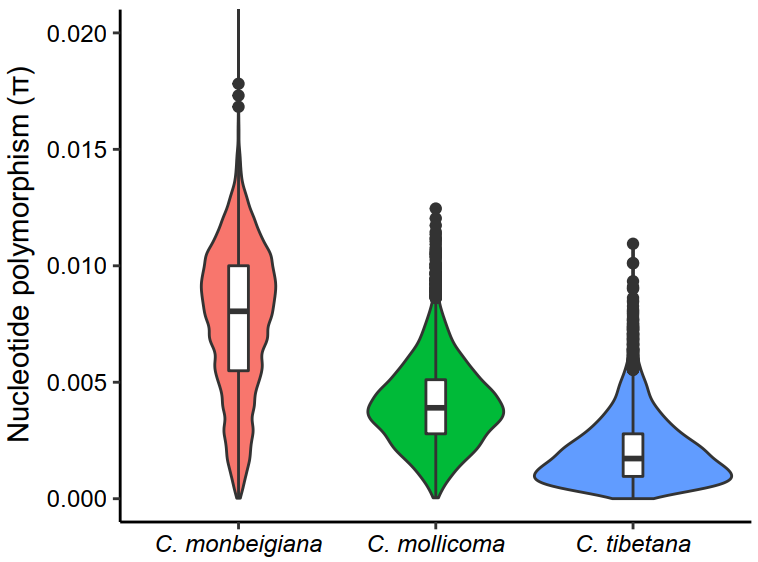


## Figure S5. Comparisons of Nucleotide polymorphism (π) between *C. monbeigiana*, *C. mollicoma* and *C. tibetana* using 50-kb sliding windows.


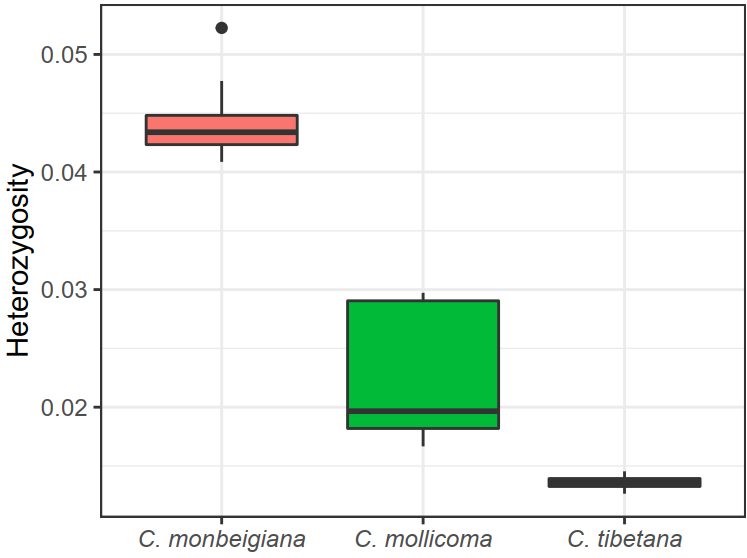


## Figure S6. Individual heterozygosity for *C. monbeigiana*, *C. mollicoma* and *C. tibetana*. Boxes indicate the 25-75% percentiles, bars indicate medians, and the whiskers extend to 1.5 x the interquartile range.


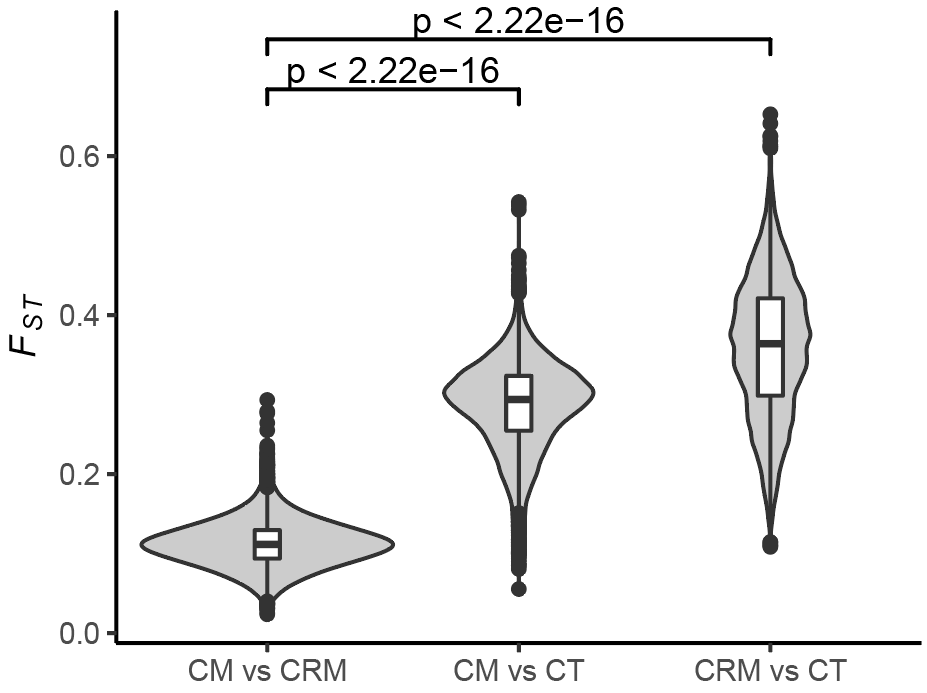


## Figure S7. Pairwise genetic divergence (*F_ST_*) in 50-kb sliding windows for *C. monbeigiana* (CM), *C. mollicoma* (CRM) and *C. tibetana* (CT) comparisons. *P*-value were calculated using t-test.


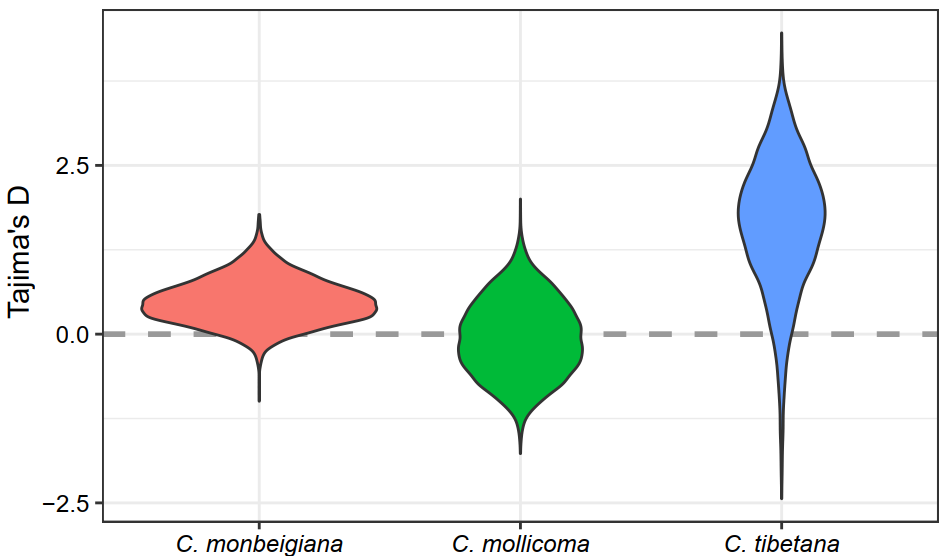


## Figure S8. Comparisons of Tajima’s D between *C. monbeigiana*, *C. mollicoma* and *C. tibetana*.


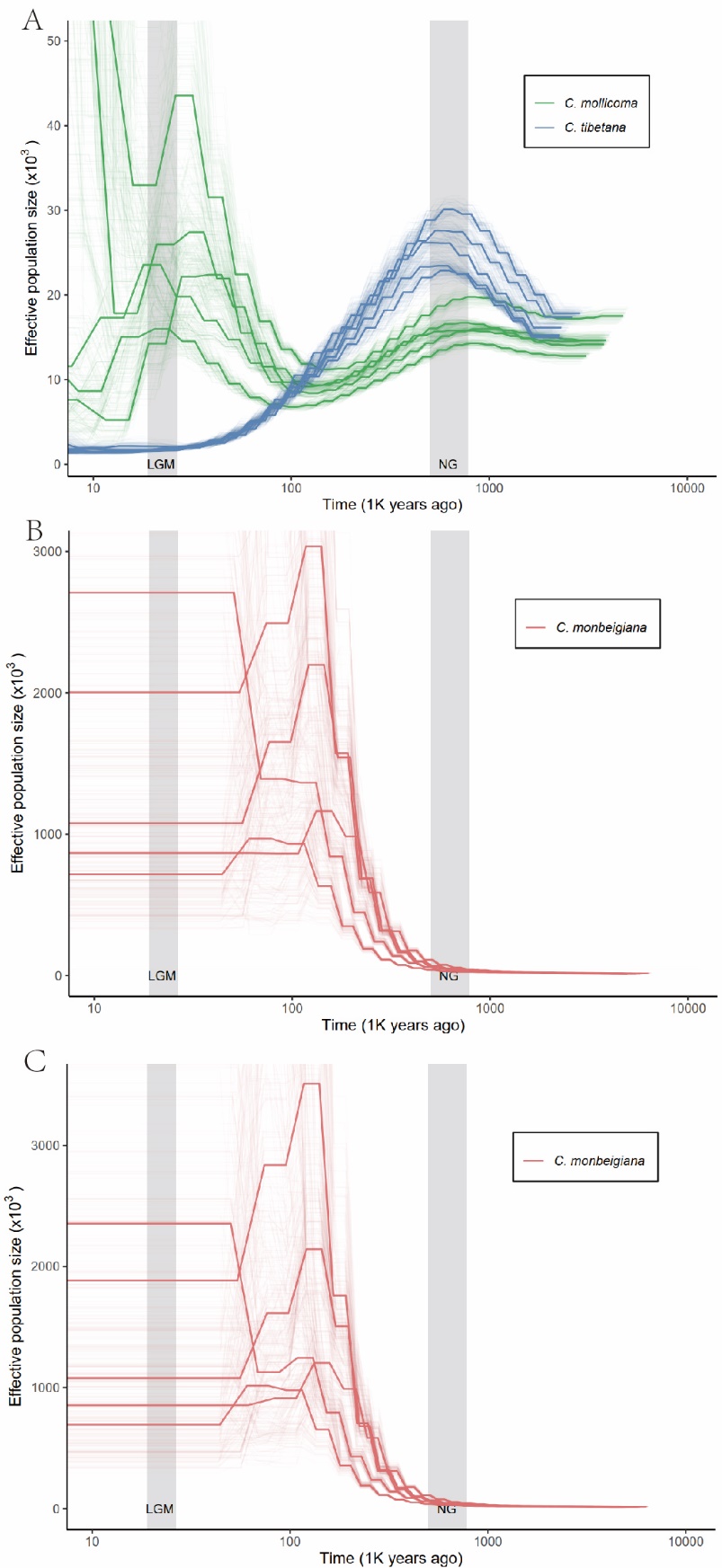


## Figure S9. Inferred demographic history for *C. monbeigiana, C. mollicoma* and *C. tibetana* and by PSMC using all sites (A, B) and non-selection sites (C). Changes in effective population size (*Ne*) through time inferred by the Pairwise Sequentially Markovian Coalescent model. The thick line is the estimated history, whereas the thin transparent line represents the 100 bootstrap runs. The period of the last glacial maximum (LGM, ~20 kya) and the Naynayxungla Glaciation (NG, 780–500 kya) are shaded in grey.


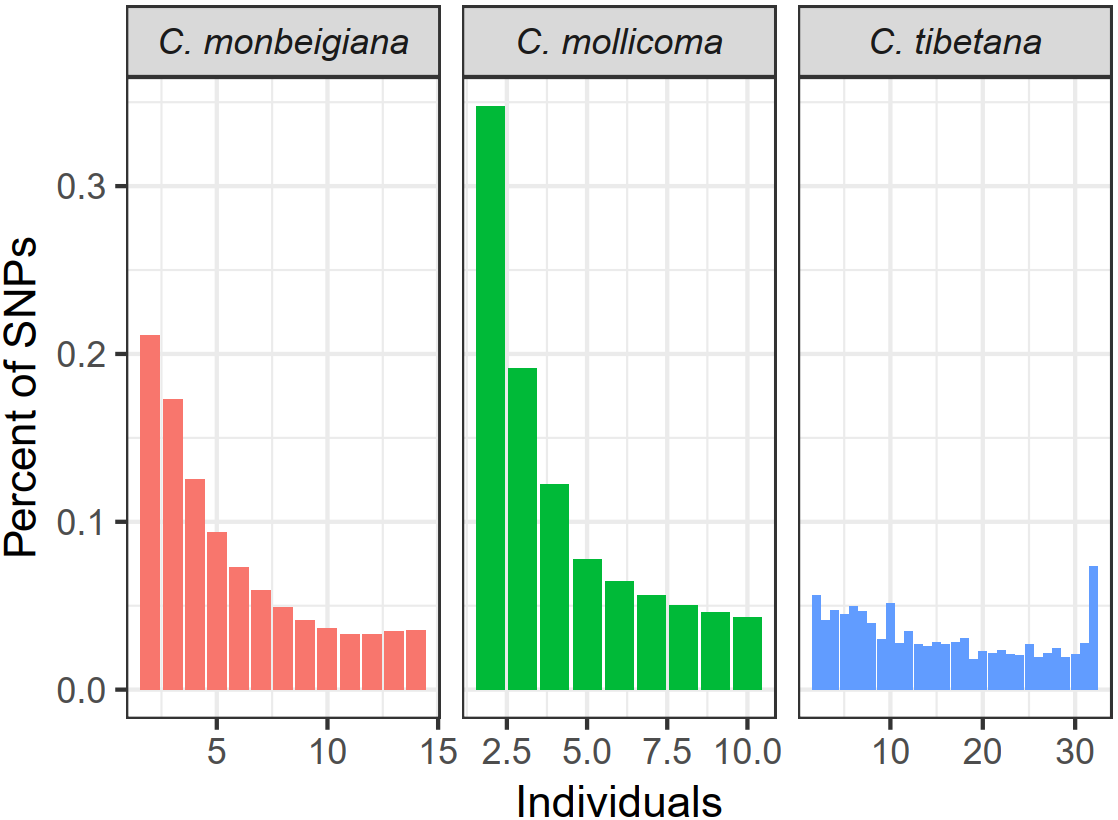


## Figure S10. Comparisons of Folded site frequency spectrum of C. monbeigiana, C. mollicoma and C. tibetana.


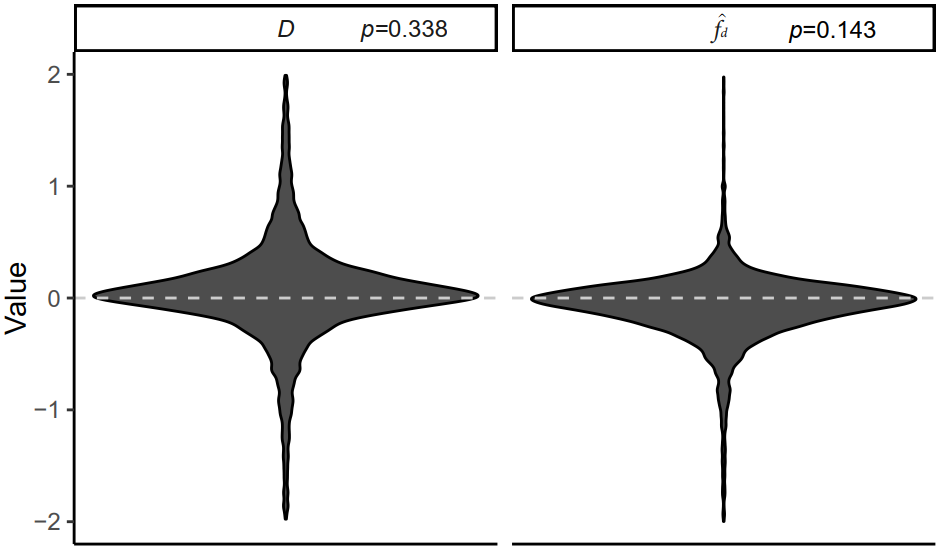


## Figure S11. ABBA-BABA statistics for *C. monbeigiana*, *C. mollicoma* and *C. tibetana* and using *C. cordata* and *Ostrya japonica* as outgroup (see model in Figure S2).


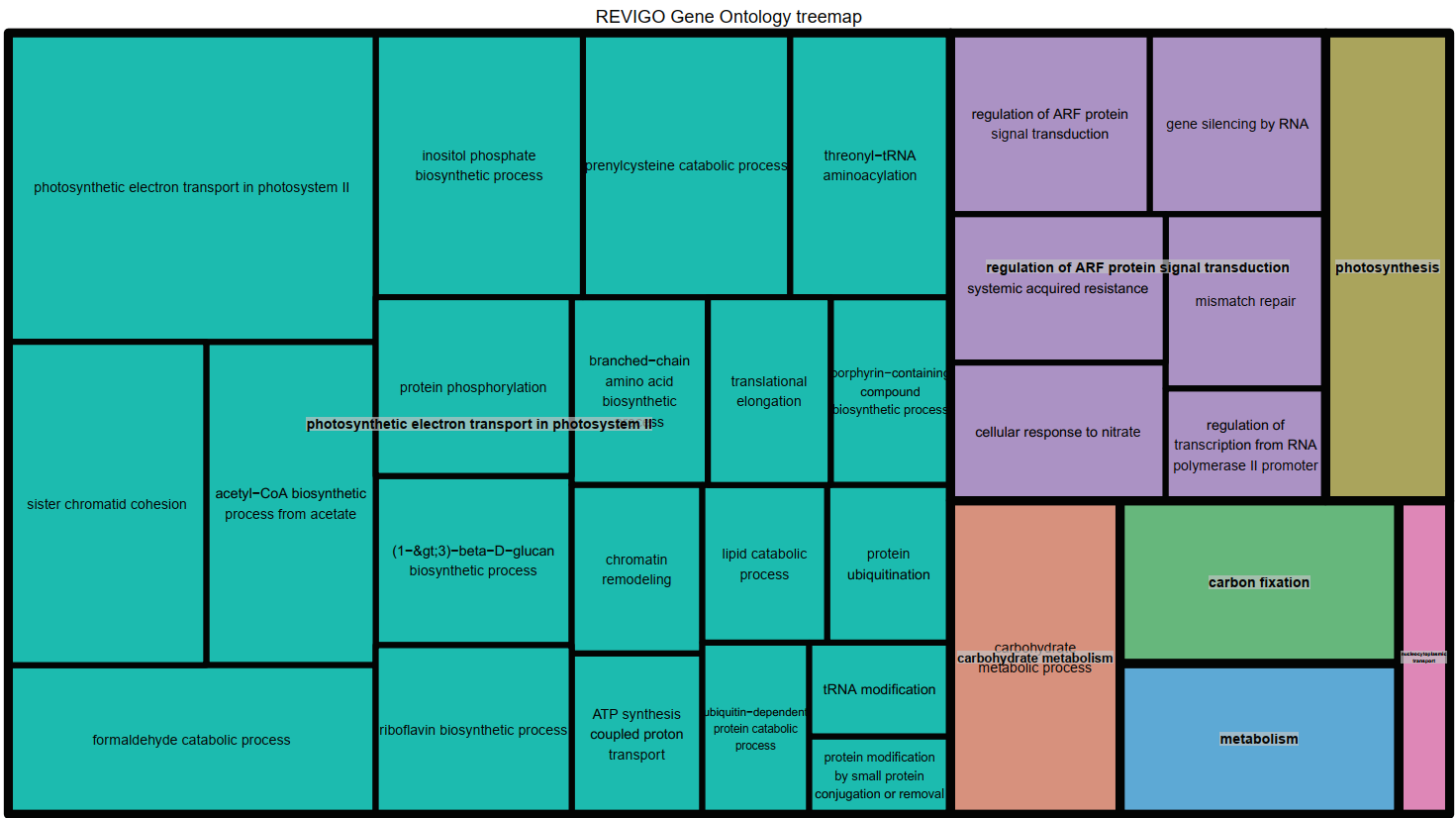


## Figure S12. Gene ontology enrichment analysis of significantly (*P*<0.05) differentiated SNPs with a 50-kb sliding window of *F_ST_*.


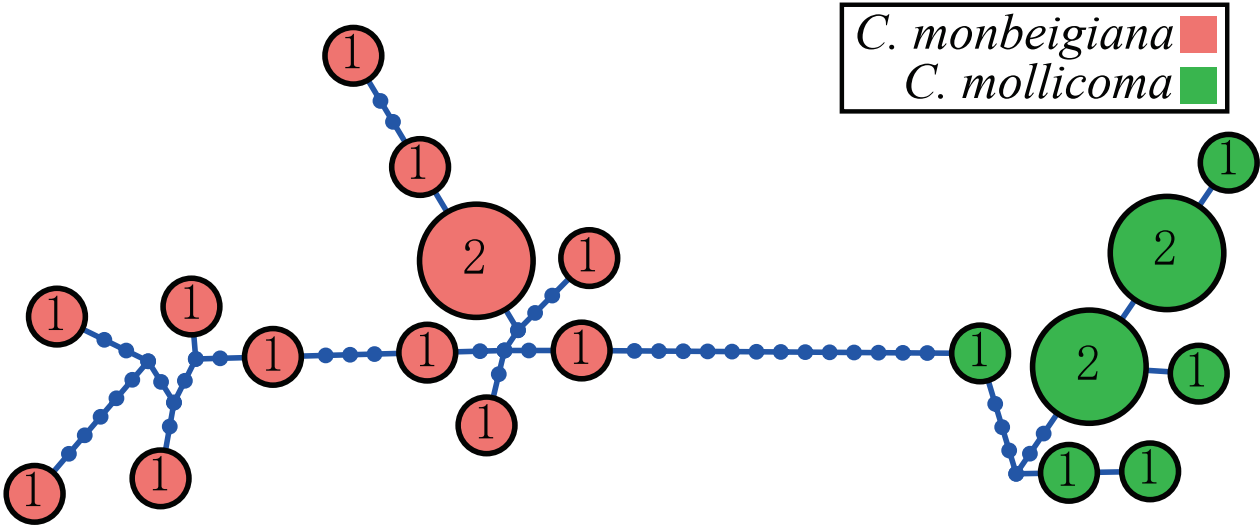


## Figure S13. Median-joining network of *Cfa015116* within *C. monbeigiana* and *C. mollicoma*.

Reference:

Durand, E. Y., Patterson, N., Reich, D., and Slatkin, M. (2011). Testing for ancient admixture between closely related populations. *Mol. Biol. Evol.* 28, 2239–2252. doi:10.1093/molbev/msr048.

Yang, X., Wang, Z., Zhang, L., Hao, G., Liu, J., and Yang, Y. (2020). A chromosome-level reference genome of the hornbeam, Carpinus fangiana. *Sci. Data*. doi:10.1038/s41597-020-0370-5.
